# Correction to: Extracellular Vesicles Derived from Neural Progenitor Cells––a Preclinical Evaluation for Stroke Treatment in Mice

**DOI:** 10.1007/s12975-021-00961-x

**Published:** 2021-11-09

**Authors:** X. Zheng, L. Zhang, Y. Kuang, V. Venkataramani, F. Jin, K. Hein, M. P. Zafeiriou, C. Lenz, W. Moebius, E. Kilic, D. M. Hermann, M. S. Weber, H. Urlaub, W.-H. Zimmermann, M. Bähr, Thorsten R. Doeppner

**Affiliations:** 1 Department of Neurology, University Medical Center Goettingen, Robert-Koch-Str. 40, 37075 Goettingen, Germany; 2 Institute of Pathology, University Medical Center Goettingen, Goettingen, Germany; 3 Department of Hematology, Cancer Center, The First Hospital of Jilin University, Changchun, Jilin China; 4 Institute for Pharmacology and Toxicology, University Medical Center Goettingen, Goettingen, Germany; 5 DZHK (German Center for Cardiovascular Research), partner site Goettingen, Göttingen, Germany; 6 Cluster of Excellence “Multiscale Bioimaging: from Molecular Machines to Networks of Excitable Cells” (MBExC), University of Goettingen, Göttingen, Germany; 7 Bioanalytical Mass Spectrometry Group, Max Planck Institute for Biophysical Chemistry, Goettingen, Germany; 8 Institute of Clinical Chemistry, Bioanalytics, University Medical Center Goettingen, Goettingen, Germany; 9 Department of Neurogenetics, Electron Microscopy Group, Max Planck Institute of Experimental Medicine, Goettingen, Germany; 10 Regenerative and Restorative Medical Research Center, Istanbul Medipol University, Istanbul, Turkey; 11 Department of Neurology, University of Duisburg-Essen Medical School, Essen, Germany; 12 Institute for Neuropathology, University Medical Center Goettingen, Goettingen, Germany

**Correction to: Translational Stroke Research**

**https://doi.org/10.1007/s12975-020-00814-z**

In the version of this article initially published, there were errors on pages 5 and 6. The protein concentration for the low, medium, and high EV groups should be 10 μg, 100 μg, and 1000 μg instead of 1 μg, 10 μg, and 100 μg.

