# Supplementary material for: Extracellular Vesicles Derived from Neural Progenitor Cells––a Preclinical Evaluation for Stroke Treatment in Mice
Source: Transl Stroke Res. 2020 May 2;12(1):185–203. doi: 10.1007/s12975-020-00814-z (PMC7803677; doi:10.1007/s12975-020-00814-z)
Supplement: Supplementary file 2 — (PDF 1737 kb) [file 12975_2020_814_MOESM2_ESM.pdf]

Translational Stroke Research

Extracellular vesicles derived from neural progenitor cells—a preclinical evaluation for stroke treatment in mice

--Manuscript Draft--

**Manuscript Number:**	TRSR-D-19-00341R2
**Full Title:**	Extracellular vesicles derived from neural progenitor cells—a preclinical evaluation for stroke treatment in mice
**Article Type:**	Original Article
**Keywords:**	cerebral ischemia; extracellular vesicles, neural progenitor cells; neurological recovery; neuroregeneration
**Corresponding Author:**	Xuan Zheng<br>Georg-August-Universitat Gottingen Universitatsmedizin<br>GERMANY
**Corresponding Author Secondary Information:**	
**Corresponding Author's Institution:**	Georg-August-Universitat Gottingen Universitatsmedizin
**Corresponding Author's Secondary Institution:**	
**First Author:**	Xuan Zheng
**First Author Secondary Information:**	
**Order of Authors:**	Xuan Zheng
	Lin Zhang
	Yaoyun Kuang
	Vivek Venkataramani
	Fengyan Jin
	Katharina Hein
	Maria Patapia Zafeiriou
	Christof Lenz
	Wiebke Möbius
	Ertugrul Kilic
	Dirk M Hermann
	Martin S Weber
	Henning Urlaub
	Wolfram-Hubertus Zimmermann
	Mathias Bähr
	Thorsten R Doeppner
**Order of Authors Secondary Information:**	

Funding Information:

TÜBITAK	Prof. Thorsten R Doeppner
Deutsche Forschungsgemeinschaft (DE) (EXC 2067/1- 390729940)	Dr. Maria Patapia Zafeiriou
Deutsche Forschungsgemeinschaft (DE) (ZI 708/10-1; CRC 1002 C04 and S01; IRTG1816 RP12; EXC 2067/1- 390729940)	Prof. Wolfram-Hubertus Zimmermann

Powered by Editorial Manager® and ProduXion Manager® from Aries Systems Corporation

	German Center for Cardiovascular Research (DZHK)	Prof. Wolfram-Hubertus Zimmermann
	Fondation Leducq (14CVD04)	Prof. Wolfram-Hubertus Zimmermann

**Abstract:**	Stem cells such as mesenchymal stem cells (MSCs) enhance neurological recovery in preclinical stroke models by secreting extracellular vesicles (EVs). Since previous reports have focused on the application of MSC-EVs only, the role of the most suitable host cell for EV enrichment and preclinical stroke treatment remains elusive. The present study aimed to evaluate the therapeutic potential of EVs derived from neural progenitor cells (NPCs) following experimental stroke. Using the PEG technique, EVs were enriched and characterized by electron microscopy, proteomics, rt-PCR, nanosight tracking analysis, and Western blotting. Different dosages of NPC-EVs displaying a characteristic profile in size, shape, protein cargo, and non-coding RNA contents were incubated in the presence of cerebral organoids exposed to oxygen-glucose-deprivation (OGD), significantly reducing cell injury when compared to control organoids. Systemic administration of NPC-EVs in male C57BL6 mice following experimental ischemia enhanced neurological recovery and neuroregeneration for as long as three months. Interestingly, the therapeutic impact of such NPC-EVs was found to be not inferior to MSC-EVs. Flow cytometric analyses of blood and brain samples seven days post-stroke demonstrated increased blood concentrations of B and T lymphocytes after NPC-EV delivery, without affecting cerebral cell counts. Likewise, a biodistribution analysis after systemic delivery of NPC-EVs revealed the majority of NPC-EVs to be found in extracranial organs such as the liver and the lung. This proof-of-concept study supports the idea of EVs being a general concept of stem cell-induced neuroprotection under stroke conditions, where EVs contribute to reverting the peripheral post-stroke immunosuppression.

Powered by Editorial Manager® and ProduXion Manager® from Aries Systems Corporation

Cover letter

Click here to access/download;Manuscript;Cover_letter_Revision_II.docx

Dear Professor Zhang,

Thank you for the encouraging response from your side. We have made the corrections as suggested by the two reviewers. Plus, we have still found some additional minor mistakes, which we have now corrected, too.

Thank you for considering our work for publication in Translational Stroke Research.

Best regards from Germany,

Thorsten R. Döppner, M.D., M.Sc.

Professor of Neurology

Reviewer #1

1.) I am glad to see that the authors have included a power calculation, I am wondering if this calculation has taken into account the mice that had to be excluded. In other words, because some of the mice died did this influence the power?

We also did a post power calculation (excluded the dead mice), whose results showed the following: the power was 0.9822147 for both behavioral test analysis and immunofluorescence analysis, and the effect size was 0.5784490.

2.) Although figures seem to be of higher quality, I still lack some detail. Whether this is in the power of the authors to fix or a compression issue of the pdf after submission I am not sure.

Unfortunately, this is indeed a consequence of the PDF compression. The original TIFF files do not have this problem. There is a link on each figure within the PDF file. This should lead you to the original TIFF. The same is also true for the main body of the paper; the word file shows some layout changes after PDF conversion as well.

Reviewer #3

1.) In Figure 8, please clarify to which delivery route and condition representative immunofluorescence images refer.

This information is now given in the revised figure legend.

2.) In the Figure 8 legend please add the information what is the scale bar.

This information is now given in the revised figure legend as well.

Manuscript
Click here to access/download;Manuscript;Revised_Manuscript_Revision_II.

Extracellular vesicles derived from neural progenitor cells—a preclinical evaluation for stroke treatment in mice

Running title: NPC-derived EVs protect against stroke

Zheng X[1], Zhang L[1], Kuang Y[1], Venkataramani V[2], Jin F[3], Hein K[1], Zafeiriou MP[4,5,6], Lenz C[7,8], Moebius W[9], Kilic E[10], Hermann DM[11], Weber MS[1,12], Urlaub H[7,8], Zimmermann W-H[4,5,6], Bähr M[1], and Doeppner TR[1,10]

[1]University Medical Center Goettingen, Department of Neurology, Goettingen, Germany

[2]University Medical Center Goettingen, Institute of Pathology, Goettingen, Germany

[3]The First Hospital of Jilin University, Department of Hematology, Cancer Center, Changchun, Jilin, China

[4]University Medical Center Goettingen, Institute for Pharmacology and Toxicology, Goettingen, Germany

[5]DZHK (German Center for Cardiovascular Research), partner site Goettingen, Germany

[6]Cluster of Excellence "Multiscale Bioimaging: from Molecular Machines to Networks of Excitable Cells" (MBExC), University of Goettingen, Germany

[7]Max Planck Institute for Biophysical Chemistry, Bioanalytical Mass Spectrometry Group, Goettingen, Germany

[8]University Medical Center Goettingen, Institute of Clinical Chemistry, Bioanalytics, Goettingen, Germany

[9]Max Planck Institute of Experimental Medicine, Electron Microscopy Group, Department of Neurogenetics, Goettingen, Germany

[10]Istanbul Medipol University, Regenerative and Restorative Medical Research Center, Istanbul, Turkey

[11]University of Duisburg-Essen Medical School, Department of Neurology, Essen, Germany

[12]University Medical Center Goettingen, Institute for Neuropathology, Goettingen, Germany

Correspondence to: Thorsten R. Doeppner, M.D.-M.Sc.

Dept. of Neurology, University Medical Center Goettingen

Robert-Koch-Str. 40, 37075 Goettingen, Germany

Phone: +49-551-3966660, fax: +49-551-399348

email: thorsten.doeppner@med.uni-goettingen.de

Keywords:

cerebral ischemia; extracellular vesicles, neural progenitor cells; neurological recovery; neuroregeneration

Abstract

Stem cells such as mesenchymal stem cells (MSCs) enhance neurological recovery in preclinical stroke models by secreting extracellular vesicles (EVs). Since previous reports have focused on the application of MSC-EVs only, the role of the most suitable host cell for EV enrichment and preclinical stroke treatment remains elusive. The present study aimed to evaluate the therapeutic potential of EVs derived from neural progenitor cells (NPCs) following experimental stroke. Using the PEG technique, EVs were enriched and characterized by electron microscopy, proteomics, rt-PCR, nanosight tracking analysis, and Western blotting. Different dosages of NPC-EVs displaying a characteristic profile in size, shape, protein cargo, and non-coding RNA contents were incubated in the presence of cerebral organoids exposed to oxygen-glucose-deprivation (OGD), significantly reducing cell injury when compared to control organoids. Systemic administration of NPC-EVs in male C57BL6 mice following experimental ischemia enhanced neurological recovery and neuroregeneration for as long as three months. Interestingly, the therapeutic impact of such NPC-EVs was found to be not inferior to MSC-EVs. Flow cytometric analyses of blood and brain samples seven days post-stroke demonstrated increased blood concentrations of B and T lymphocytes after NPC-EV delivery, without affecting cerebral cell counts. Likewise, a biodistribution analysis after systemic delivery of NPC-EVs revealed the majority of NPC-EVs to be found in extracranial organs such as the liver and the lung. This proof-of-concept study supports the idea of EVs being a general concept of stem cell-induced neuroprotection under stroke conditions, where EVs contribute to reverting the peripheral post-stroke immunosuppression.

Introduction

The systemic transplantation of stem cells such as mesenchymal stem cells (MSCs) and neural progenitor cells (NPCs), promotes neurological recovery, angiogenesis, and neurogenesis in animal models of cerebral ischemia [1-7]. The majority of these grafted cells, however, are trapped in extracerebral organs and do not integrate into existing neural networks. Rather, transplanted stem cells display both low survival rates and poor differentiation rates within the ischemic milieu [8-11], suggesting an indirect mode of action by which post-stroke neurological recovery is achieved.

It has been demonstrated that stem cell-derived conditioned medium induces similar effects in various disease models when compared to stem cell transplantation itself [12, 13]. MSCs and other cell types secrete neurotrophic factors such as EGF and VEGF, thus inducing both neuroprotection and neuroregeneration [14-16]. Recent research, however, has questioned the hypothesis that these beneficial factors are the sole biological key mediator of stem cell-induced brain protection against cerebral ischemia. Instead, bilayer structured vesicles, termed extracellular vesicles (EVs), secreted from eukaryotic cells, including MSCs, have been found to be critical players in the aforementioned process. These EVs have also been detected in conditioned medium derived from stem cells [17, 18], further supporting the idea that EVs are biological mediators of stem cell-induced actions under conditions of cerebral ischemia.

EVs are a heterogeneous group of vesicles ranging in size from 30 nm to 1000 nm. They contain a defined set of cargo, which depends on the characteristics of the source cell [19]. EV cargo consists of non-coding RNAs, DNA, and proteins such as heat shock proteins and tetraspanins [20, 21]. Although fundamental questions in the emerging EV field still need to be addressed, the current concept divides EVs into exosomes, microvesicles, and apoptotic bodies. Current stroke research focuses on microvesicles and especially exosomes. Both exosomes and microvesicles are a consequence of direct outward budding or pinching of the plasma membrane, with microvesicles being derivates of multivesicular bodies (MVB) that bud on the cell surface to be released into the extracellular environment [22]. The present work

focuses on further elucidating the therapeutic potential of EVs rather than on distinguishing the various subtypes of EVs. Hence, the term EVs is used throughout the manuscript.

EVs exert a plethora of beneficial therapeutic effects in various disease models such as myocardial ischemia, liver fibrosis, kidney injury, and cerebral ischemia [23-27]. Indeed, previous research from our group and others have systematically studied the impact of systemic MSC-derived EV infusion on stroke outcome, demonstrating increased neurological recovery, enhanced neuroregeneration, and a modified post-stroke immune response upon EV treatment [17, 28, 29]. Whereas the majority of regenerative stroke research focuses on the transplantation of MSCs, therapeutic results were also observed for other cell types such as NPCs [1, 30-32]. On the contrary, the therapeutic potential of subventricular zone-derived NPC-EVs under stroke conditions has not been addressed. Although EVs appear to be an attractive tool for prospective adjuvant stroke treatment, fundamental issues have to be solved, amongst which is the relevance of the host cell for EV harvest. Therefore, following in vitro characterization of enriched NPC-EVs, we evaluated their therapeutic potential in models of both in vitro hypoxia and in vivo stroke.

Materials and Methods

Primary culture of MSCs and NPCs

P1 newborn mice were anesthetized using CO_2 euthanasia. The brain was removed, and the subventricular zone (SVZ) was dissected in cold PBS under microscopic control. Tissue chunks were spun down at 200 g for 1 min at 4°C. The supernatant was discarded, and the tissue was incubated with 1 ml of 0.05 % Trypsin-EDTA in 15-ml conical tubes. The tubes were gently shaken at room temperature (RT) for 15 min. Each cell pellet was resuspended with 5.5 ml of NPC cell culture medium (DMEM F-12 medium, B27 (Thermofisher, Waltham, USA), L-glutamine (Thermofisher, Waltham, USA), 1x Pen-Strep (Thermofisher, Waltham, USA), 20 ng/ml of FGF-2 (Thermofisher, Waltham, USA), and 20 ng/ml EGF (Thermofisher, Waltham, USA)) to which 5.5 ml of Percoll/PBS solution was added. The tubes were mixed by inversion. Thereafter, another centrifugation step with 400 g was performed for 15 min at RT. The cell pellet was washed three times with 10 ml NPC medium and spun down at 200 g for 5 min at RT each time to collect the cells. Finally, the pellet was washed once more with 8 ml of NPC medium. The cell pellet was resuspended with 1 ml of DMEM-F12, and the cells were then plated onto 24 cm^2 cell culture plates. The cells were cultured in a 5 % CO_2 incubator. The neurospheres were observed within 72 h. On day 3, growth factors (20 ng/ml of FGF-2 and 20 ng/ml EGF) were added to the cell culture. The cell passage period of NPCs was 5 to 6 days.

MSCs obtained from allogeneic adipose tissue of C57BL/J mice (25-30 g) were cultured. The adipose tissue was digested with collagenase (Sigma-Aldrich, St. Louis, USA). Primary MSCs were cultured in a T75 flask. Each flask contained $3.6x10^6$ cells incubated under standard cell culture condition (37ºC, 5% CO_2) in MSC culture medium (DMEM F-12 medium, fetal bovine serum (FBS, Thermofisher, Waltham, USA), 1x Pen-Strep (Thermofisher, Waltham, USA)). The cell passage period of MSCs was 6 to 7 days.

EV enrichment from cultured NPCs and MSCs

After passage 3, NPCs were treated with Accutase (Sigma-Aldrich, St. Louis, USA) and transferred to T75 cell culture flasks with 30 ml NPC culture medium without growth factors. Each T75 contained 36x10^6 NPCs. A total of 12 of T75 cell culture flasks was used in each EV isolation, meaning EVs from 432x10^6 cells were isolated. NPC-conditioned medium (NPC-CM) was collected after 24 h of incubation under standard cell culture conditions. Large vesicles and debris were removed by filtration through 220 nm pore filters (TPP Techno Plastic Products AG, Trasadingen, Switzerland). The NPC-CM was kept frozen (-80°C) until further processing. After the thawing of the NPC-CM, EVs were enriched using the polyethylene glycol (PEG) precipitation method, as previously described [19, 33]. In brief, PEG precipitation was performed at a final concentration of 10% PEG 6,000 (50% wt/vol; Merck Group, Darmstadt, Germany) and 75 mM NaCl. After incubation for 12 h at 4°C, the EVs were concentrated by centrifugation for 45 min at 4,500 g. EV pellets were resuspended in saline to a total volume of 30 ml and precipitated by ultracentrifugation for 2 h at 110,000 g (Optima XPN-80 Ultracentrifuge, Beckman Coulter, Brea, USA). The target speed was 30,000 U/min and both the acceleration and the brake were set to maximum. EV pellets were resuspended and diluted in saline to a concentration of 500 µl containing EVs obtained from CM of 432x10^6 NPCs. Aliquots of 500 µl each were stored at -80°C until usage. The MSC-EV isolation was done after the third MSC passage. MSCs were cultured overnight with an FBS-free culture medium and after 24 h, the cell supernatants were collected. EVs were obtained from these supernatants by using the same protocol as for the NPC-EV PEG isolation method.

Since the optimal enrichment procedure for NPC-EVs is still a matter for debate [19], we applied differential centrifugation, i.e., ultracentrifugation only, for some experiments. As such, some NPC-EV samples were treated with ultracentrifugation as described before without the application of PEG 6,000. In brief, the cell culture medium was filtered through 220 nm filters to remove cell debris and apoptotic bodies followed by a 2 h ultracentrifugation procedure at 110,000 g.

Nanoparticle tracking analyses (NTA)

For both size determination and quantification of enriched NPC-EVs, an NTA was performed using the Nanosight platform (NanoSight LM10, Malvern Panalytical, Kassel, Germany). As shown previously [34], 1:1,000 water-diluted samples were measured in duplicate, and 400 µl of the diluted sample was injected into the measurement chamber. Each sample was measured three times, and the length of the video of each measurement was set to 30 s.

Western blot analysis

Protein concentrations of EV samples were determined using the micro-bicinchoninic acid assay (Thermofisher, Waltham, USA). Western blots were performed with 5 µg of the concentrated EV fractions, which were treated with sample buffer (dithiothreitol, 0.1 % SDS, 0.1 M Tris HCl; pH 7.0) and boiled for 5 min at 95°C before separation on 12 % SDS-polyacrylamide gel electrophoresis gels. The samples were transferred to polyvinylidene fluoride membranes (Merck Group, Darmstadt, Germany). The membranes were blocked by 5 % milk solution (skim milk powder (Thermofisher, Waltham, USA) dissolved in Tris-buffered saline solution with 1 % Tween-20) for 1 h at RT and stained with antibodies recognizing "exosomal marker" proteins including CD63 (1:500, Biorbyt, Cambridge, UK), TSG101 (1:500, GeneTex, Irvine, USA), Alix (1:500, BD Transduction Laboratories, San Jose, USA), and negative control calnexin (1:500, Abcam, Cambridge, UK) followed by a 1 h incubation with a matched horseradish peroxidase-labeled secondary antibody. Immunoreactivity was detected using chemiluminescence detection kit reagents and a Chemidoc Station (Biorad, Hercules, USA). Image J was used for densitometric analysis of each blot. The Western blotting procedures were repeated three times per sample. Please also refer to Supplementary Figure S2 for uncropped Western blots.

Transmission electron microscopy (TEM)

TEM was used to investigate the microstructure of NPC-EVs. Briefly, formvar-coated TEM grids (copper, 150 hexagonal mesh, Science Services, Munich, Germany) were put on

the top of a droplet of the respective EV fraction and incubated for 10 min. Then, the grids were washed five times by incubation for 2 min in PBS, followed by similar incubations with ultra-pure water. For contrast, the grids were incubated for 5 min on droplets of uranylacetate-oxalate, followed by 5 min incubation on droplets of a 1:9 dilution of 4 % uranylacetate in 2 % methylcellulose. These solutions were prepared as described as before [35]. After draining the methylcellulose from the grids using filter paper and drying of the methylcellulose film as previously described, samples were imaged with a LEO912 transmission electron microscope (Carl Zeiss Microscopy, Oberkochen, Germany) and images were taken using an on-axis 2k CCD camera (TRS-STAR, Stutensee, Germany).

Mass spectrometric analyses

NPC-EV samples were reconstituted in 1x NuPAGE LDS Sample Buffer (Invitrogen, Carlsbad, USA) and separated on 4-12 % NuPAGE Novex Bis-Tris Minigels (Invitrogen, Carlsbad, USA). Gels were stained with Coomassie Blue for visualization purposes and each lane sliced into 12 equidistant lanes regardless of staining. After washing, gel slices were reduced with dithiothreitol (DTT), alkylated with 2-iodoacetamide and digested with trypsin overnight. The resulting peptide mixtures were then extracted, dried in a SpeedVac, reconstituted in 2 % acetonitrile/ 0.1 % formic acid/ (v:v) and prepared for nanoLC-MS/MS as described previously [36].

For mass spectrometric analysis samples were enriched on a self-packed reversed phase-C18 precolumn (0.15 mm ID x 20 mm, Reprosil-Pur120 C18-AQ 5 µm, Dr. Maisch, Ammerbuch-Entringen, Germany) and separated on an analytical reversed phase-C18 column (0.075 mm ID x 200 mm, Reprosil-Pur 120 C18-AQ, 3 µm, Dr. Maisch, Ammerbuch-Entringen, Germany) using a 30 min linear gradient of 5-35 % acetonitrile/ 0.1 % formic acid (v:v) at 300 nl min^{-1}). The eluent was analyzed on a Q Exactive hybrid quadrupole/orbitrap mass spectrometer (ThermoFisher Scientific, Dreieich, Germany) equipped with a FlexIon nanoSpray source and operated under Excalibur 2.4 software using a data-dependent acquisition method. Each experimental cycle was of the following form: one full MS scan

across the 350-1600 m/z range was acquired at a resolution setting of 70,000 FWHM, and the AGC target of $1x10^6$ and a maximum fill time of 60 ms. Up to the 12 most abundant peptide precursors of charge states 2 to 5 above a $2x10^4$ intensity threshold were then sequentially isolated at 2.0 FWHM isolation width, fragmented with nitrogen at a normalized collision energy setting of 25 %, and the resulting production spectra recorded at a resolution setting of 17,500 FWHM, and AGC target of $2x10^5$ and a maximum fill time of 60 ms. Selected precursor m/z values were then excluded for the following 15 s. Two technical replicates per sample were acquired. Peak lists were extracted from the raw data using Raw2MSMS software v1.17 (Max Planck Institute for Biochemistry, Martinsried, Germany). Protein identification was achieved using MASCOT 2.5.1 software (Matrixscience, London, UK). Proteins were identified against the UniProtKB mouse reference proteome v2017.09 (16930 protein entries) along with a set of 51 contaminants commonly identified in our laboratory. The search was performed with trypsin as enzyme and iodoacetamide as a cysteine blocking agent. Up to two missed tryptic cleavages and methionine oxidation as a variable modification were allowed for. Search tolerances were set to 10 ppm for the precursor mass, 0.05 Da for fragment masses, and ESI-QUAD-TOF specified as the instrument type. For further information about the mass spectrometric analyses, please refer to supplementary material “mass spectrometric analyses” attachment.

NPC-EV RNA isolation and qRT-PCR

In order to investigate whether or not NPC-EVs contain distinct sets of miRNAs that might be responsible for the biological effects of EVs on neuroprotection or neuroregeneration, we chose several miRNA candidates according to the literature [37-41]. Total RNA, including miRNAs, was extracted using TRIzol (Invitrogen, Carlsbad, USA) according to the manufacturer’s instructions. ANanoDropND1000 spectrophotometer (NanoDrop, Wilmington, DE, USA) was used to measure the RNA concentrations. The KAPA SYBR® FAST One-Step Kit for LightCycler®480 (Merck Group, Darmstadt, Germany) was used to perform qRT-PCR according to the manufacturer’s instruction request. The PCR primers were purchased from

Eurofins Genomics and U6 as an internal control. miR-124: forward 5'-GCGAGGATCTGTGAATGCCAAA-3' and reverse 5'-AGATGGTGATGGGCTTCCC-3'. miR-145: forward 5'-GUCCAGUUUUCCCAGGAAUCCCU-3' and reverse 5'-GGAUUCCUGGGAAAACUGGACUU-3'. miR-17: forward 5'-GAGCCAAAGTGCTTACAGTGC-3' and reverse 5'-AGTGCAGGGTCCGAGGTATT-3'. miR-19b: forward 5'-GGGCAAATCCATGCAAAAC-3' and reverse 5'-AGTGCAGGGTCCGAGGTATT-3'. miR-20a: forward 5'-TGGGTAAAGTGCTTATAGTGC-3' and reverse 5'-AGTGCAGGGTCCGAGGTATT-3'. miR-26a: forward: 5'-CCGCCGTTCAAGTAATCCAG-3' and reverse 5'-AGTGCAGGGTCCGAGGTATT-3'. miR-26b: forward 5'-CGCCGCTTCAAGTAATTCAGGAT-3' and reverse 5'-GTGCAGGGTCCGAGGT-3'. miR-23a: forward 5'- CAGGCGGGTAGTAGATG-3' and reverse 5'-AGGGACGGGCATGGAAAGG-3'. miR-126: forward 5'-CGCGCCGTACCGTGAGTAA-3' and reverse 5' -GTGCAGGGTCCGAGGT-3'. U6: forward 5'-TCGCTTCGGCAGCACATA-3' and reverse 5'-GGGCCATGCTAATCTTCTCTG-3'. The PCR cycling included reverse transcription stage at 42°C for 5 min and 95°C for 3 min followed by amplification stage at 95°C for 10 s and 58°C for 20 s. At the melting curve stage, the temperature was set to 95°C for 5 s followed by 65°C for 1 min and set the acquisition mode to continuous, duration mode set to 5-10 acq/°C, the temperature was 97°C at the end. The cooling stage was set to 40°C for 10 s. miRNA expression was quantified using the $2^{-\Delta Ct}$ method

Cerebral organoids and oxygen-glucose deprivation (OGD assay)

Cerebral organoids were generated from in-house generated iPSCs (hiPS-G1) [42] embedded in a collagen hydrogel [Zafeiriou et al., in revision]. Directed differentiation was performed by dual smad inhibition (SB/noggin) in the presence of retinoic acid and subsequently supported by FGF-2, TGF-beta1, and DAPT exposure. Cerebral organoids on culture day 30 were used for analyses. Before the induction of OGD, cerebral organoids were cultured in standard cortex culture medium in 24-well plates for seven days. Thereafter, cerebral organoids were washed with PBS twice before induction of the OGD, using a

commercially available hypoxia chamber (Toepffer Laborsysteme GmbH, Göppingen, Germany). For the induction of OGD, the cerebral organoids were incubated in glucose-free BSS0 solution under hypoxic conditions for 8-10 h. The OGD chamber setting was as follows: 37°C, O_2 less than 0.5 %, CO_2 5%, and humidity 70%. The amount of NPC-EVs applied to organoids was calculated by the following process: the EVs from $432x10^6$ NPCs were diluted in 500 µl of PBS, each microlitre contained $8.64x10^5$ cell equivalent EVs ($8.64x10^5$ cell equivalent/µl, 43.2 µg/µl). Three different NPC-EV concentrations were tested: NPC-EVs low (EVs equivalent to $2x10^5$ NPCs, i.e., 1 µg of NPC-EVs), NPC-EVs medium (EVs equivalent to $2x10^6$ NPCs, i.e., 10 µg of NPC-EVs), NPC-EVs high (EVs equivalent to $2x10^7$ NPCs, i.e., 100 µg of NPC-EVs). EVs were carefully vortexed for 1 min before applying to organoids in order to prevent any aggregation of EVs. After hypoxia, the organoids were taken out from the hypoxia chamber followed by 24 h of reoxygenation with normal cell culture medium in an incubator (5 % CO_2) under standard cell culture conditions. The EVs were added to the organoids at the beginning of the OGD and at the beginning of the reoxygenation. Terminal deoxynucleotidyl transferase dUTP nick end labeling (TUNEL, In Situ Cell Death Detection Kit, Merck Group, Darmstadt, Germany) staining was used according to the manufacturer guidance in order to detect cell death rates, and 4′,6-diamidino-2-phenylindole (DAPI) staining was used in order to stain nuclei.

Experimental paradigm and animal groups

No human samples were used for the study. All studies were performed with governmental approval according to the NIH guidelines for the care and use of laboratory animals. Both the STAIR criteria and the ARRIVE guidelines were followed. Male C57BL6 mice aged 10 weeks (Harlan Laboratories, Darmstadt, Germany) were kept under circadian rhythm with free access to food and water. The mice were randomly assigned to the treatment groups. At all stages of the study, the researchers were blinded from the experimental conditions chosen. Middle cerebral artery occlusion (MCAO) was induced. Animals were anesthetized at 2% to 2.5% isoflurane during the MCAO surgery. A heating pad set to 37.0 °C

was put under the animal to keep the body temperature at normal. The left common carotid artery (LCCA), left external carotid artery (LECA) and left internal carotid artery (LICA) were carefully dissected from the surrounding nerves and tissues. A permanent ligation was made on the LCCA, LECA, and a free knot was also made on the LICA. A silicon-coated monofilament (Doccol Corp., Sharon, USA) was inserted into the LCCA and then gently pushed forward towards the offspring of the left middle cerebral artery (MCA) through a small hole which was cut on the LCCA until a the significant drop of the blood flow on the laser Doppler flow (LDF) recording was observed. During the experiment, the LDF was recorded with a flexible probe (Perimed AB, Järfälla, Sweden) above the core of the left MCA territory. A drop in the blood flow of more than 80 % to the baseline was considered to indicate successful surgery. Sixty minutes after monofilament insertion, the reperfusion was initiated by monofilament removal, and the LDF recordings were continued for an additional 15 min before the wounds were carefully sutured. The amount of NPC-EVs applied to organoids was calculated by the following process. The EVs from 432×10^6 NPCs were diluted to 500 µl with PBS, and each microlitre contains 8.64×10^5 cell equivalent EVs (8.64×10^5 cell equivalent/µl, 43.2 µg/µl). The mice were exposed to MCAO followed by administration of normal saline (control), NPC-EVs medium (EVs equivalent 2×10^6 NPCs, 10 µg), NPC-EVs low (EVs equivalent 2×10^5 NPCs, 1 µg), or NPC-EVs high (EVs equivalent 2×10^7 NPCs, 100 µg), MSC-EVs medium (EVs equivalent 2×10^6 MSCs, 10 µg), MSC-EVs low (EVs equivalent 2×10^5 MSCs, 1 µg), or MSC-EVs high (EVs equivalent 2×10^7 MSCs, 100 µg). The result of the power calculation was 0.8372017 for both behavioral test analysis and immunofluorescence analysis, assuming an effect size of 0.4399022. EVs were carefully vortexed for 1 min before injection in order to prevent aggregation of EVs. The latter was suspended in 100 µl PBS before injection. The administration occurred 24 h after the stroke via right femoral vein injection. The procedure was repeated for all experimental groups (saline and EV groups) on day 3 and on day 5 post-stroke using retroorbital injection techniques in order to avoid bilateral femoral vein injections. All samples were injected at a rate of 250 µl over 10 min. The mice were allowed to survive for a maximum of 84 days. These mice were used for the behavioral analyses and

the immunofluorescence staining studies of brain injury and neurorestoration. Precise numbers of animals used are given for each condition in the figure legends and in the Supplementary Table S1 including survival rates of mice.

In vivo biodistribution study of NPC-EVs

Three animals per group were used for the evaluation of EV biodistribution after different administration methods and conditions (femoral vein injection in sham mice, femoral vein injection after MCAO, retroorbital injection in sham mice, and retroorbital injection after MCAO). The EVs were labeled with Celltracker CM-DiI (Invitrogen, Carlsbad, USA) according to the manufacturer's instruction. In brief, DiI was incubated with EVs (EV equivalent $2x10^6$ NPCs, 10 µg) at 37°C for 1 h. In the MCAO group, labeled EVs were injected into mice after MCAO surgery. Mice were sacrificed 2 h after the injection. Cryosections (14 µm thick) of the brain, lung, and the liver were counterstained with 4',6-diamidino-2-phenylindole (DAPI), and the biodistribution was analyzed by immunofluorescence staining. Images were acquired using an Axioplan 2 imaging e microscope (Carl Zeiss AG, Hombrechtikon, Germany). Mean fluorescence intensity was measured by Image J (version 1.80).

Flow Cytometry

Single-cell suspensions were prepared for flow cytometry. The mice were exposed to MCAO followed by administration of normal saline (control), NPC-EVs low (EVs equivalent to $2x10^5$ NPCs, 1 µg), NPC-EVs medium (EVs equivalent to $2x10^6$ NPCs, 10 µg) or NPC-EVs high (EVs equivalent to $2x10^7$ NPCs, 100 µg) at day 1,3,5 after surgery. The power calculation of the flow cytometry analysis yielded 0.8243373 with an assumed effect size of 0.8596798. The mice were sacrificed by an overdose of isoflurane at day 7 after surgery. Blood samples were collected into EDTA-coated tubes by puncture of the inferior vena cava followed by transcranial perfusion with ice-cold 0.1 M PBS and brain removal. Erythrocytes were lysed by incubation with lysis buffer (155 mM NH_4Cl, 10 mM $KHCO_3$, 3 mM EDTA) for 5 min followed by two washing steps with 0.1 M PBS. The ischemic part of the brain was dissected, followed

by centrifugated and separated using a Percoll-gradient. Leukocytes and inflammatory cells were isolated from the intermediate phase. Cell suspension from brain and blood were blocked with Fc-block (CD16/32 FcX rat anti-mouse IgG, BioLegend, San Diego, USA) to interrupt non-specific binding and afterwards stained for CD45 (CD45 rat anti-mouse IgG- brilliant Violett 510, BD Horizon, Franklin Lakes, USA), CD3 (CD3 rat anti-mouse IgG- PE, BD Horizon, Franklin Lakes, USA), CD19 (CD19 rat anti-mouse IgG-APC, BD Horizon, Franklin Lakes, USA) and CD11b (CD11b rat anti-mouse IgG-PE- Cy7, eBioscience, Darmstadt, Germany). For data analysis, FlowJo v10.0 software was used. Please also refer to Supplementary Table S2 and Supplementary Figure S1 for further information.

Analysis of post-stroke motor coordination deficits

The mice were trained on days 1 and 2 before the induction of stroke to ensure proper test behavior. The tests for analysis of motor coordination were performed at the time points given using the tight rope test, the balance beam test, and the corner turn test, as previously described [43]. The tight rope test and the balance beam test were performed three times on each test day, and the mean values were calculated. For the balance beam test, the readout parameter was the time until the mice reached the platform, with a maximal testing time of 60 s. Assessment of the tight rope test results was done using a validated score ranging from 0 (minimum) to 20 (maximum). The corner turn test included 10 trials per test day, during which the laterality index (number of left turns / 10) was calculated. The details of the tight rope test score sheet can be found in Supplementary Table S3.

Immunofluorescence staining

Brain injury as indicated by neuronal density was evaluated in 16 µm cryostat sections stained with a mouse monoclonal anti-NeuN antibody (1:500; Merck Group, Darmstadt), which was detected by a goat anti-mouse Alexa Fluor 488 antibody (Invitrogen, Carlsbad, USA). For analysis, the regions of interests (ROIs) were defined at anterior-posterior +0.14 mm, medial-lateral ±1.15 to 2.25 mm, and dorsal-ventral -2.25 to 3.25 mm. Five sections per mouse were

analyzed, and in each section, five ROIs were examined. The mean neuronal densities were determined for all ROIs. Endogenous cell proliferation and differentiation of new-born cells were analyzed after a single daily i.p. injection of 5-Bromo-2-deoxyuridine (BrdU; 50 mg/kg body weight; Sigma-Aldrich, St. Louis, USA) from days 40 to 84. The sections were counterstained for BrdU and doublecortin (Dcx; immature neuronal marker) and NeuN (mature neuronal marker). A monoclonal mouse anti-BrdU (1:400; Roche Diagnostics, Basel, Switzerland), a monoclonal rat anti-BrdU (1:400; Abcam, Cambridge, UK), a polyclonal goat anti-Dcx (1:50; Santa Cruz Biotechnology, Heidelberg, Germany), and a monoclonal mouse anti-NeuN (1:350; Merck Group, Darmstadt, Germany) were used. All primary antibodies were detected using appropriate Cy-3-labeled or Alexa Fluor 488-labeled secondary antibodies (Jackson Immuno, West Grove, USA) followed by DAPI staining.

For axonal plasticity, the anterograde tract tracer biotinylated dextran amine (BDA) was applied by stereotactic injection into the contralateral non-impaired cortex on day 70 post-stroke. As such, mice were deeply anesthetized and fixed into a stereotactic frame (ASI instruments, SAS-4100, Warren, USA). BDA was injected using a Hamilton syringe at 0.5 mm rostral from bregma, 2.5 mm lateral from the midline, and 1.5 mm ventral from the cortical surface. The syringe was kept in place for an additional 5 min after the end of the injection. A BDA detection kit (Lifetechnologies, Carlsbad, USA) was used after the sacrifice of the animals following the manufacturer's instructions.

Statistical analysis

For comparison of two groups, the two-tailed independent Student's t-test was used. For comparison of three or more groups, a one-way analysis of variance (ANOVA) followed by the Tukey's post-hoc-test and, if appropriate, a two-way ANOVA were used. G*Power was used to calculate the power of the experiment and GraphPad Prism was used for statistics. Unless otherwise stated, data are presented as mean with SD values. A p-value of <0.05 was considered statistically significant.

Results

Characterization of NPC-EVs

Since the optimal procedure for EV enrichment remains uncertain [19], we systematically analyzed NPC-EVs using both the PEG method and ultracentrifugation only. The subsequent characterization of such enriched NPC-EVs included transmission electron microscopy (TEM), nanosight tracking analysis (NTA), mass spectrometry, and Western blotting. Western Blot analysis for selected EV biomarkers revealed that CD63, TSG101, TAPA1, and Alix were present in NPC-EVs obtained from both PEG enrichment and ultracentrifugation only (Fig. 1A-B). No difference was observed with regard to quantitative analysis of these proteins.

NTA revealed the distribution of NPC-EVs to be in the range of 30 nm to 300 nm, which is typical of exosomes and microvesicles alike (Fig. 1C). The concentration between the two isolation methods was similar ($18x10^9$ in the PEG method compared to $15x10^9$ in the ultracentrifugation only method). Along with the NTA, experiments using TEM revealed a typical EV morphology (Fig. 1D). Furthermore, no other types of vesicles were found in our samples, suggesting that NPC-EVs were predominantly exosomes and microvesicles.

By using mass spectrometric analyses, we successfully detected proteins which are crucial for EV biogenesis or are known to be associated with stimulated angiogenesis and neurogenesis such as HSP70, both in PEG-enriched and in ultracentrifugation-enriched NPC-EVs (please also refer to the supplementary section). EV cytosolic proteins, transmembrane proteins, or GPI-anchored proteins, which are crucial for EV biogenesis, such as ANXA6, SDCPB, HSPA8, TSG101, CD81, and CD9 were found in the EV samples (Fig. 2A-B). There was no significant difference between the two groups with respect to cytosolic or transmembrane proteins. Although calnexin was considered as a negative control for EVs, calnexin can also be found in some subtypes of exosomes [19]. Other subtypes of exosomal markers such as GM130 and CYC1 were close to the detection threshold or not detectable at all (Fig. 2C). Furthermore, strict EV negative markers (as purity control) such as APOA1, UMOD, and ALB were close to the detection threshold or not detectable at all in PEG or the

ultracentrifugation group (Fig. 2D), suggesting a sufficiently high level of purification in EVs enriched with either PEG or ultracentrifugation.

Since miRNAs support angiogenesis, neurogenesis, and neuroprotection [44], we chose some of those miRNA candidates which have been identified as beneficial in the aforesaid aspects. Indeed, typical miRNA candidates were found in NPC-derived EVs, with the subtypes miR-20a, miR-26b, and miR-124 being at the highest concentrations (Fig. 2E). Conversely, miR-133, and miR-145 were hardly found in NPC-derived EVs at all. The isolation method, however, did not significantly affect these miRNA levels in NPC-EVs.

Since both enrichment procedures did not significantly differ between each other for the majority of the readout parameters analyzed for NPC-EV characterization, further experiments were performed using the PEG method only. In this context, the PEG approach is more feasible than ultracentrifugation, allowing the handling of large volumes of conditioned medium with ease.

NPC-EVs protect cerebral organoids from oxygen-glucose-deprivation *(OGD)*

After having characterized the aforementioned EVs, we then established an in vitro model of OGD in cerebral organoids, which better reflect the physiological situation than neuronal monolayer cultures do. We first tested the time course of OGD-induced cell death of cerebral organoids by exposing the organoids to either 8 h or 10 h of OGD, followed by 24 h of reoxygenation under standard cell culture conditions (Fig. 3A-B). Cell death rates under these conditions were significantly increased in organoids exposed to both 8 h and 10 h of OGD when compared to cerebral organoids that were kept under standard culture conditions (Fig. 3B). However, no significant difference was found between the two OGD groups themselves. Accordingly, we chose an OGD exposure of 8 h for the following experiments. Cerebral organoids were then treated with low, medium or high concentrations of NPC-derived EVs, and the cell death rate was measured after 8 h of OGD followed by 24 h of reoxygenation (Fig. 3C). Indeed, exposure to all three concentrations of NPC-EVs significantly reduced the cell death rate of cerebral organoids under these conditions, as assessed by TUNEL staining.

Delivery of NPC-EVs reduces post-ischemic motor coordination impairment

In light of the aforementioned in vitro data on cerebral organoids, we tested the hypothesis that NPC-EVs improve neurological recovery after cerebral ischemia in mice. Following a previously published protocol on MSC-EVs [17], NPC-EVs of different dosages (low, medium, and high) were systemically administered on days 1, 3, and 5 post-stroke, with MSC-EVs serving as internal controls (Fig. 4A-C). Administration of a medium dosage of both NPC-EVs and MSC-EVs resulted in significantly better test performance of these animals in the tight rope test as well as in the corner turn test when compared to controls (Fig. 4B-C). Of note, NPC-EVs were not inferior to MSC-EVs, and the better test performance of mice treated with either NPC-EVs or MSC-EVs was long-lasting and thus stable until the end of the observation period of 84 days. In the balance beam test, however, the beneficial effects of NPC-EVs and MSC-EVs delivered at a medium dose were only transiently effective (Fig. 4A). Delivery of NPC-EVs or MSC-EVs at low or high dosages only partially induced neurological recovery in these three tests, if at all. The laser Doppler flow was used to ensure the quality of the MCAO model in each group. Each group showed a significant blood flow drop during the surgery, and there was no significant difference between the various treatment groups (Fig. 4D).

Neurological recovery does not necessarily imply an effect on brain tissue injury or brain regeneration and vice versa. We subsequently analyzed neuronal survival in the ischemic striatum at 84 days after the stroke. In line with the reduction of neurological impairment, increased neuronal densities were found in mice treated with medium doses of both NPC-EVs or MSC-EVs (Fig. 5A), again showing no difference between these two groups. Low and high doses of NPC-EVs or MSC-EVs were not effective. Conclusively, NPC-derived EVs reduce post-stroke brain injury on both the histological and the functional level and are not inferior to MSC-derived EVs. It is important to note that the therapeutic effect in EV formulation was highly dose dependent.

NPC-EV delivery stimulates post-stroke neuroregeneration and axonal plasticity

As stated before, neurological recovery is not always associated with histological changes, and the mechanisms that lead to enhanced neurological recovery are diverse. In this context, we hypothesized that NPC delivery might stimulate endogenous repair mechanisms of the brain, including increased levels of neurogenesis. Taking into account that neurogenesis also takes place in the adult mammalian brain, with endogenous stem cells being stimulated upon induction of cerebral ischemia, the application of NPC-EVs might positively interfere with this process. Indeed, analysis of the cell proliferation marker BrdU showed significantly increased levels of BrdU$^+$ cells (Fig. 5B) in the NPC-EV medium group. The co-expression analysis with the proliferation marker BrdU and the neuronal marker NeuN revealed increased levels of NeuN$^+$/BrdU$^+$ cells on day 84 within the ischemic striatum of animals treated with a medium dosage of NPC-EVs (Fig. 5C). On the contrary, the relative amount of BrdU$^+$ cells expressing the immature neuronal marker Dcx was not affected by NPC-EV-treatment (Fig. 5D).

Neuroregeneration is a complex process that is not only limited to neurogenesis. We therefore investigated the extent of axonal plasticity on day 84 in the post-ischemic brain (Fig. 6), using contra-lateral stereotactic injections of BDA in the non-impaired cortex. Again, delivery of a medium dose of NPC-EVs but not low or high doses of NPC-EVs significantly enhanced axonal plasticity in these mice when compared to the control group (Fig. 6). Treatment with a medium dosage of MSC-EVs yielded similar effects with regard to neuroregeneration and axonal plasticity as observed for NPC-EVs, suggesting again that NPC-EVs are not inferior to MSC-EVs.

NPC-EVs reverse peripheral post-stroke immunosuppression

The pathophysiology of cerebral ischemia comprises of a complex string of diverse inflammatory signaling cascades, not solely being harmful to the surrounding ischemic tissue [45]. In our previous study, MSC-EVs did not affect the immune response in the central nervous system but reversed the post-ischemic immunosuppression in the peripheral blood

seven days after stroke. In NPC-EVs, we saw similar effects. Consequently, NPC-EV treatment of mice with either dosage did not affect leukocytes ($CD45^{high}$), monocytes ($CD45^{high}CD3^{-}CD11b^{+}$), B cells ($CD45^{high}CD3^{-}CD19^{+}$) or T cells ($CD45^{high}CD3^{+}$) within the ischemic CNS (Fig. 7A-D). Flow cytometry analysis in the blood of mice treated with a medium but not a low or high dosage of NPC-EVs revealed significantly increased levels of both B lymphocytes and T lymphocytes when compared to the control group (Fig. 7E-H).

NPC-EVs predominantly distribute in peripheral organs

The majority of MSCs and other transplanted cells do not reach the brain, but are trapped in extracranial organs [46]. Even though EVs are known to pass the blood-brain barrier [47], the fact that NPC-EVs predominantly modulated the peripheral but not the central immune system (Fig. 8) might suggest that the majority of EVs do not reach the brain, either. Since different administration methods and conditions might affect the biodistribution patterns of NPC-EVs, we compared two different delivery routes, i.e., femoral vein injection and retroorbital injection under both ischemic and non-ischemic conditions. The biodistribution of NPC-EVs was similar in different methods and different conditions (Fig. 8). NPC-EVs were not only found in peripheral organs such as the liver and the lung but also found in the brain. However, most of these EVs were detected in the liver and in the lung when compared to the brain. There was no difference between the liver and the lung with regard to NPC-EV biodistribution patterns.

Discussion

Although EVs have recently been recognized as potential therapeutic tools in the treatment of stroke [48], previous work has almost exclusively focused on the application of MSC-derived EVs only [17]. As such, the relevance of the stem cell source for EV enrichment remains elusive, although recent data on pluripotent stem cell-derived NSC-EVs has become available [49, 50]. The present study elucidated whether or not EVs have a cell-type independent therapeutic potential against stroke that is not restricted to MSC-derived EVs. The latter will be of high therapeutic relevance under clinical stroke conditions in order to choose proper tissue sources or pooled fractions from cell sources beyond MSCs or related cells. Since both endogenous and grafted SVZ-derived NPCs contribute to neurological recovery and neuroregeneration upon experimental stroke [51], EVs from such SVZ-derived NPCs have been enriched in the present study. We provide evidence for NPC-EVs not only increasing cell resistance against hypoxic injury in vitro, but also for enhancing post-stroke neuroregeneration and neurological recovery in vivo. Applying a direct comparison against MSC-EVs, for the first time we demonstrate that SVZ-derived NPC-EVs exhibit a similar therapeutic activity as MSC-EVs in a mouse model of stroke.

In light of various EV enrichment procedures, i.e., ultracentrifugation, precipitation, chromatography, or density gradient separation [52], the optimal enrichment technique is still a matter of debate [19]. The pros and cons of each technique have to be thoroughly balanced when being applied. In that respect, precipitation methods using PEG or others offer quick and easy handling of large cell supernatant volumes for EV enrichment, although none of these techniques currently qualify for EV enrichment under GMP standards. Previous work from our own group systematically analyzed the PEG precipitation approach in direct comparison to standard EV enrichment procedures on HEK293T cells [33]. The latter revealed the PEG approach to be not inferior to standard EV enrichment procedures using HEK293T cells. Compared to the ultracentrifugation method, the PEG method can concentrate a high volume of conditioned medium in low centrifugation force (4,500 g) which can reduce EV damage from shear force during the ultracentrifugation process. Although basic EV properties such as the

size should be unaffected when dealing with different cell sources [53], the optimal concentrations of these cell sources under different isolation methods remains a matter of debate. To the best of our knowledge, PEG precipitation has not been used for the enrichment of SVZ-derived NPC-EVs. In order to exclude an impact of PEG precipitation on NPC-derived EVs, a detailed characterization of the latter was therefore performed.

Using PEG 6000, we successfully isolated EVs from NPC conditioned medium. The purification rates obtained by this method were high for NPC-EVs, as indicated in the mass spectrometric analysis result. Especially, EV negative markers such as APOA1, UMOD, and ALB were close to the detection threshold or not detectable at all in our PEG method group and ultracentrifugation only group. Likewise, the distribution patterns were similar to EVs enriched with ultracentrifugation only, which is still regarded as a gold standard [19]. Our observations are in line with previous work from our group on the comparison of the PEG method and direct ultracentrifugation or differential centrifugation, indicating that PEG does not significantly affect the purity of EVs [33], although artifacts and aggregations still occur as observed in the TEM analysis. As a matter of fact, previous studies on EV application under pathological conditions such as Alzheimer's disease [54] and stroke [55], have either focused on exosomes only or used the general term of EVs, which might be more convenient in light of a therapeutic approach. The mass spectrometric analyses on NPC-EVs as performed in the present work indicated expression patterns of both exosomal markers and microvesicle markers [19]. Some of these proteins found in our EV samples are well-known key mediators of neuroprotection and neurogenesis. The heat shock protein HSP70, for instance, was highly abundant in NPC-derived EVs. HSP70 has frequently been described to mediate a plethora of signaling cascades, all of which contribute to an enhanced resistance of neural cells under hostile hypoxic or ischemic conditions [56]. Under such circumstances, HSP70 has been shown to modify oxidative stress and proteasomal activity of cerebral tissue exposed to hypoxic or ischemic injury, resulting in enhanced cell survival and increased neurological recovery of stroke rodents [56]. The increased resistance of cerebral organoids exposed to

OGD injury treated with NPC-EVs might, therefore, be at least partially mediated by proteins such as HSP70 and others.

Since pluripotent stem cell-derived NSC-EVs have recently been proven to increase tissue rescue and functional outcomes in translational murine and porcine stroke models [49, 50], our data support the hypothesis that EVs from different cell sources including SVZ-derived NPCs could induce neurological recovery as well. As indicated before, EVs do not only carry diverse sets of proteins but also contain DNA and non-coding RNA, among which are miRNAs of particular interest. The latter has been extensively studied under physiological and pathological conditions alike, not only related to cerebral ischemia where they serve both as biomarkers and therapeutic tools [57]. Screening for selected miRNAs to be likely expressed in NPC-derived EVs revealed enhanced levels of miR-124, which is among the most abundant miRNAs in the adult mammalian brain [58, 59]. MiR-124 affects a plethora of signaling molecules such as the recently identified inhibition of deubiquitination of Usp14, significantly contributing to the reduction of post-stroke brain injury in rodents [60]. Increased levels of miR-124 and others such as the microRNA 17-92 cluster found in NPC-EVs, known to contribute to neuroprotection or enhanced neuroregeneration [61-67], might thus further enhance the resistance of cells and tissues against hypoxia or ischemia. Since EVs are able to transfer cargo like miRNAs to target cells [68], it is fair enough to hypothesize that NPC-EVs yield protection of cerebral organoids and brain tissue using this way of action. In this context, recent research has demonstrated that oligodendrocytes are able to transfer mitochondria toward neurons via microvesicles upon induction of hypoxia, a mechanism that might also be of relevance under the conditions chosen for the present work [69-71]. Elucidating such a precise mechanism was, however, beyond the scope of the present work which set emphasis on the therapeutic potential of NPC-EVs rather than on mechanisms involved in such a process.

The therapeutic potential of stem cells and the different mechanisms being involved greatly depend on delivery routes and transplantation timing [3]. Indeed, systemic delivery of MSCs or NPCs under experimental stroke conditions reduces post-stroke brain injury, enhances neurological recovery, and stimulates neuroregenerative processes [3, 72, 73].

Systemic delivery of MSC-derived EVs, likewise, mediates the aforementioned effects in a similar fashion [17]. Both timing and the delivery route were chosen following a previously established protocol for the intravenous application of MSC-derived EVs [17], although future studies might enhance the therapeutic time frame for EVs even further. Contrary to grafted stem cells, which predominantly do not reach the brain and which involve possible side effects such as neoplasia [74], EVs pass the blood-brain barrier [75]. Recent work has demonstrated that systemic injection of exosomes results in intracerebral enrichment of siRNA derived from these ectopic exosomes [76]. Consequently, the systemic injection of NPC-derived EVs is likely to induce neurological recovery and promote neuroregeneration via enrichment of both cargo proteins and non-coding RNAs after passing of the blood-brain barrier by ectopic EVs. Interestingly, NPC-EVs did not affect post-stroke central immune responses, which is in line with our data acquired on the application of MSC-EVs. Rather, NPC-EVs reverse the post-stroke immunosuppression, as was observed for MSC-EVs. In detail, NPC-EVs increased both peripheral T and B cell numbers, with the latter being known to support neurogenesis and functional recovery after stroke [77]. The idea of a peripheral way of action of systemically delivered NPC-EVs is further backed up by the fact that labeled NPC-EVs predominantly end up in peripheral organs such as the lung and the liver rather than in the brain. However, additional studies regarding homing and targeting of ectopic EVs are required to elucidate these underlying mechanisms further.

In our study, medium dosages of NPC-EVs (and also MSC-EVs) showed optimal outcomes when compared to either low or high dosages of EVs. Although we do not have clear evidence, it appears to be feasible that high dosages might lead to the formation of embolisms, resulting in the early death of animals. On the contrary, low dosages of EVs might result in an insufficient concentration of either cargo proteins or non-coding RNAs, thus failing to induce neuroprotection or neuroregeneration. However, the medium dosages of NPC-EVs and MSC-EVs only showed transiently beneficial effects in the balance beam test, which might be due to the lower sensitivity of the balance beam test when compared to the tight rope test and the corner turn test at later stages of the disease [78, 79]. As such, high sensitivity is of

uttermost importance, especially at chronic stages of the disease. Studies on the dose-dependent effects of EVs are surprisingly short, but Tabak et al. indeed suggest that EVs affect target tissues in a dose-dependent manner [80]. Thus, the present work also indicates that future studies should pay attention to the EV dosage chosen when planning both pre-clinical investigations or clinical trials.

In conclusion, the present study demonstrates that SVZ-derived NPC-EVs are effective as therapeutic tools under experimental stroke conditions. The underlying mechanisms appear to be pleiotropic and complex but are in part due to a modulation of peripheral post-stroke immune responses. In light of NPC-EVs being non-inferior to MSC-EVs, the idea of EVs as general therapeutic tools independent of cell sources is thus further supported. As a matter of fact, the tremendous translational potential of EVs is evident, with distinct advantages when compared to stem cell transplantation. After a myriad of negative clinical trials in the field of neuroprotective drugs against stroke, EVs might revolutionize modern stroke treatment as an available tool next to clinically established re-canalization strategies.

Compliance with Ethical Standards

Funding

This study was funded by TÜBITAK (to T.R.D.). P.M.Z. is funded by the Deutsche Forschungsgemeinschaft (DFG, German Research Foundation) under Germany's Excellence Strategy - EXC 2067/1- 390729940. W.H.Z. is supported by the DFG (ZI 708/10-1; CRC 1002 C04 and S01; IRTG1816 RP12; EXC 2067/1- 390729940), by the German Center for Cardiovascular Research (DZHK), and the Fondation Leducq (14CVD04).

Disclosure/conflict of interest

The authors declare to have no conflict of interest of any kind.

Ethical approval:

This article does not contain any studies with human participants performed by any of the authors. All animal experiments were performed with governmental approval according to the NIH guidelines for the care and use of laboratory animals. Both the STAIR criteria and the ARRIVE guidelines have been followed.

Acknowledgment

We thank Irina Graf and Regine Kruse (both Goettingen, Germany) for excellent technical assistance. We also thank Dr. Sarah K. Williams (Heidelberg, Germany) for proof-reading of the manuscript.

Author contribution statement

- Research and experiments were performed by Zheng, Zhang, Kuang, Venkataramani, Jin, Zafeiriou, Lenz, Moebius, Weber, and Doeppner.
- Design and concept of the study were from Zheng and Doeppner.
- Zheng, Doeppner, Hein, Hermann, Urlaub, Bähr, and Zimmermann wrote the manuscript.
- Financial support was provided by Bähr, Doeppner and Zimmermann.

References

1. Bacigaluppi, M., et al., *Delayed post-ischaemic neuroprotection following systemic neural stem cell transplantation involves multiple mechanisms.* Brain, 2009. **132**(Pt 8): p. 2239-51.

2. Banerjee, S., et al., *The potential benefit of stem cell therapy after stroke: an update.* Vasc Health Risk Manag, 2012. **8**: p. 569-80.

3. Doeppner, T.R., et al., *Effects of acute versus post-acute systemic delivery of neural progenitor cells on neurological recovery and brain remodeling after focal cerebral ischemia in mice.* Cell Death Dis, 2014. **5**: p. e1386.

4. Shen, L.H., et al., *Intracarotid transplantation of bone marrow stromal cells increases axon-myelin remodeling after stroke.* Neuroscience, 2006. **137**(2): p. 393-9.

5. Ukai, R., et al., *Mesenchymal stem cells derived from peripheral blood protects against ischemia.* J Neurotrauma, 2007. **24**(3): p. 508-20.

6. Doeppner, T.R., et al., *Transduction of Neural Precursor Cells with TAT-Heat Shock Protein 70 Chaperone: Therapeutic Potential Against Ischemic Stroke after Intrastriatal and Systemic Transplantation.* Stem Cells, 2012. **30**(6): p. 1297-310.

7. Lee, R.H., et al., *Intravenous hMSCs improve myocardial infarction in mice because cells embolized in lung are activated to secrete the anti-inflammatory protein TSG-6.* Cell Stem Cell, 2009. **5**(1): p. 54-63.

8. Darsalia, V., T. Kallur, and Z. Kokaia, *Survival, migration and neuronal differentiation of human fetal striatal and cortical neural stem cells grafted in stroke-damaged rat striatum.* Eur J Neurosci, 2007. **26**(3): p. 605-14.

9. Wei, L., et al., *Transplantation of embryonic stem cells overexpressing Bcl-2 promotes functional recovery after transient cerebral ischemia.* Neurobiol Dis, 2005. **19**(1-2): p. 183-93.

10. McDonald, J.W., et al., *Transplanted embryonic stem cells survive, differentiate and promote recovery in injured rat spinal cord.* Nat Med, 1999. **5**(12): p. 1410-2.

11. Zawada, W.M., et al., *Growth factors improve immediate survival of embryonic dopamine neurons after transplantation into rats.* Brain Res, 1998. **786**(1-2): p. 96-103.

12. Hao, P., et al., *Conditioned medium of human adipose-derived mesenchymal stem cells mediates protection in neurons following glutamate excitotoxicity by regulating energy metabolism and GAP-43 expression.* Metab Brain Dis, 2014. **29**(1): p. 193-205.

13. Timmers, L., et al., *Reduction of myocardial infarct size by human mesenchymal stem cell conditioned medium.* Stem Cell Res, 2007. **1**(2): p. 129-37.

14. Hsieh, J.Y., et al., *Mesenchymal stem cells from human umbilical cord express preferentially secreted factors related to neuroprotection, neurogenesis, and angiogenesis.* PLoS One, 2013. **8**(8): p. e72604.

15. Scheibe, F., et al., *Mesenchymal stromal cells rescue cortical neurons from apoptotic cell death in an in vitro model of cerebral ischemia.* Cell Mol Neurobiol, 2012. **32**(4): p. 567-76.

16. Tate, C.C., et al., *Human mesenchymal stromal cells and their derivative, SB623 cells, rescue neural cells via trophic support following in vitro ischemia.* Cell Transplant, 2010. **19**(8): p. 973-84.

17. Doeppner, T.R., et al., *Extracellular Vesicles Improve Post-Stroke Neuroregeneration and Prevent Postischemic Immunosuppression.* Stem Cells Transl Med, 2015. **4**(10): p. 1131-43.

18. Yang, Y., et al., *Exosomes Secreted by Adipose-Derived Stem Cells Contribute to Angiogenesis of Brain Microvascular Endothelial Cells Following Oxygen-Glucose Deprivation In Vitro Through MicroRNA-181b/TRPM7 Axis.* J Mol Neurosci, 2018. **65**(1): p. 74-83.

19. Thery, C., et al., *Minimal information for studies of extracellular vesicles 2018 (MISEV2018): a position statement of the International Society for Extracellular Vesicles and update of the MISEV2014 guidelines.* J Extracell Vesicles, 2018. **7**(1): p. 1535750.

20. Kim, D.K., et al., *EVpedia: an integrated database of high-throughput data for systemic analyses of extracellular vesicles.* J Extracell Vesicles, 2013. **2**.

21. Mathivanan, S., et al., *ExoCarta 2012: database of exosomal proteins, RNA and lipids.* Nucleic Acids Res, 2012. **40**(Database issue): p. D1241-4.

22. Doyle, L.M. and M.Z. Wang, *Overview of Extracellular Vesicles, Their Origin, Composition, Purpose, and Methods for Exosome Isolation and Analysis.* Cells, 2019. **8**(7).

23. Cantaluppi, V., et al., *Microvesicles derived from endothelial progenitor cells protect the kidney from ischemia-reperfusion injury by microRNA-dependent reprogramming of resident renal cells.* Kidney Int, 2012. **82**(4): p. 412-27.

24. Gatti, S., et al., *Microvesicles derived from human adult mesenchymal stem cells protect against ischaemia-reperfusion-induced acute and chronic kidney injury.* Nephrol Dial Transplant, 2011. **26**(5): p. 1474-83.

25. Lai, R.C., et al., *Exosome secreted by MSC reduces myocardial ischemia/reperfusion injury.* Stem Cell Res, 2010. **4**(3): p. 214-22.

26. Li, T., et al., *Exosomes derived from human umbilical cord mesenchymal stem cells alleviate liver fibrosis.* Stem Cells Dev, 2013. **22**(6): p. 845-54.

27. Xin, H., et al., *Systemic administration of exosomes released from mesenchymal stromal cells promote functional recovery and neurovascular plasticity after stroke in rats.* J Cereb Blood Flow Metab, 2013. **33**(11): p. 1711-5.

28. Joerger-Messerli, M.S., et al., *Extracellular Vesicles Derived from Wharton's Jelly Mesenchymal Stem Cells Prevent and Resolve Programmed Cell Death Mediated by Perinatal Hypoxia-Ischemia in Neuronal Cells.* Cell Transplant, 2018. **27**(1): p. 168-180.

29. Ophelders, D.R., et al., *Mesenchymal Stromal Cell-Derived Extracellular Vesicles Protect the Fetal Brain After Hypoxia-Ischemia.* Stem Cells Transl Med, 2016. **5**(6): p. 754-63.

30. Rumajogee, P., et al., *Exogenous Neural Precursor Cell Transplantation Results in Structural and Functional Recovery in a Hypoxic-Ischemic Hemiplegic Mouse Model.* eNeuro, 2018. **5**(5).

31. Wilcox, J.T., et al., *Neural precursor cell transplantation enhances functional recovery and reduces astrogliosis in bilateral compressive/contusive cervical spinal cord injury.* Stem Cells Transl Med, 2014. **3**(10): p. 1148-59.

32. Zhang, P., et al., *Human embryonic neural stem cell transplantation increases subventricular zone cell proliferation and promotes peri-infarct angiogenesis after focal cerebral ischemia.* Neuropathology, 2011. **31**(4): p. 384-91.

33. Ludwig, A.K., et al., *Precipitation with polyethylene glycol followed by washing and pelleting by ultracentrifugation enriches extracellular vesicles from tissue culture supernatants in small and large scales.* J Extracell Vesicles, 2018. **7**(1): p. 1528109.

34. Sokolova, V., et al., *Characterisation of exosomes derived from human cells by nanoparticle tracking analysis and scanning electron microscopy.* Colloids Surf B Biointerfaces, 2011. **87**(1): p. 146-50.

35. Peters, P.J. and J. Pierson, *Immunogold labeling of thawed cryosections.* Methods Cell Biol, 2008. **88**: p. 131-49.

36. Atanassov, I. and H. Urlaub, *Increased proteome coverage by combining PAGE and peptide isoelectric focusing: comparative study of gel-based separation approaches.* Proteomics, 2013. **13**(20): p. 2947-55.

37. Chen, S.R., et al., *Research on miR-126 in glioma targeted regulation of PTEN/PI3K/Akt and MDM2-p53 pathways.* Eur Rev Med Pharmacol Sci, 2019. **23**(8): p. 3461-3470.

38. Yao, L., et al., *MicroRNA-124 regulates the expression of p62/p38 and promotes autophagy in the inflammatory pathogenesis of Parkinson's disease.* FASEB J, 2019. **33**(7): p. 8648-8665.

39. Zheng, L., et al., *Overexpression of MicroRNA-145 Ameliorates Astrocyte Injury by Targeting Aquaporin 4 in Cerebral Ischemic Stroke.* Biomed Res Int, 2017. **2017**: p. 9530951.

40. Huang, B., et al., *Peptide modified mesenchymal stem cells as targeting delivery system transfected with miR-133b for the treatment of cerebral ischemia.* Int J Pharm, 2017. **531**(1): p. 90-100.

41. Xin, H., et al., *MicroRNA cluster miR-17-92 Cluster in Exosomes Enhance Neuroplasticity and Functional Recovery After Stroke in Rats.* Stroke, 2017. **48**(3): p. 747-753.

42. Tiburcy, M., et al., *Defined Engineered Human Myocardium With Advanced Maturation for Applications in Heart Failure Modeling and Repair.* Circulation, 2017. **135**(19): p. 1832-1847.

43. Balkaya, M., et al., *Assessing post-stroke behavior in mouse models of focal ischemia.* J Cereb Blood Flow Metab, 2013. **33**(3): p. 330-8.

44. Mirzaei, H., et al., *MicroRNA: Relevance to stroke diagnosis, prognosis, and therapy.* J Cell Physiol, 2018. **233**(2): p. 856-865.

45. Xing, C., et al., *Pathophysiologic cascades in ischemic stroke.* Int J Stroke, 2012. **7**(5): p. 378-85.

46. Eggenhofer, E., et al., *The life and fate of mesenchymal stem cells.* Front Immunol, 2014. **5**: p. 148.

47. Zagrean, A.M., et al., *Multicellular Crosstalk Between Exosomes and the Neurovascular Unit After Cerebral Ischemia. Therapeutic Implications.* Front Neurosci, 2018. **12**: p. 811.

48. Park, K.S., et al., *Enhancement of therapeutic potential of mesenchymal stem cell-derived extracellular vesicles.* Stem Cell Res Ther, 2019. **10**(1): p. 288.

49. Webb, R.L., et al., *Human Neural Stem Cell Extracellular Vesicles Improve Recovery in a Porcine Model of Ischemic Stroke.* Stroke, 2018. **49**(5): p. 1248-1256.

50. Webb, R.L., et al., *Human Neural Stem Cell Extracellular Vesicles Improve Tissue and Functional Recovery in the Murine Thromboembolic Stroke Model.* Transl Stroke Res, 2018. **9**(5): p. 530-539.

51. Yamashita, T., et al., *Subventricular zone-derived neuroblasts migrate and differentiate into mature neurons in the post-stroke adult striatum.* J. Neurosci., 2006. **26**(24): p. 6627-36.

52. Lane, R.E., et al., *Purification Protocols for Extracellular Vesicles.* Methods Mol Biol, 2017. **1660**: p. 111-130.

53. Haraszti, R.A., et al., *High-resolution proteomic and lipidomic analysis of exosomes and microvesicles from different cell sources.* J Extracell Vesicles, 2016. **5**: p. 32570.

54. Reza-Zaldivar, E.E., et al., *Mesenchymal stem cell-derived exosomes promote neurogenesis and cognitive function recovery in a mouse model of Alzheimer's disease.* Neural Regen Res, 2019. **14**(9): p. 1626-1634.

55. Geng, W., et al., *Exosomes from miRNA-126-modified ADSCs promotes functional recovery after stroke in rats by improving neurogenesis and suppressing microglia activation.* Am J Transl Res, 2019. **11**(2): p. 780-792.

56. Kim, J.Y., et al., *The 70-kDa heat shock protein (Hsp70) as a therapeutic target for stroke.* Expert Opin Ther Targets, 2018. **22**(3): p. 191-199.

57. Bertoli, G., C. Cava, and I. Castiglioni, *MicroRNAs: New Biomarkers for Diagnosis, Prognosis, Therapy Prediction and Therapeutic Tools for Breast Cancer.* Theranostics, 2015. **5**(10): p. 1122-43.

58. Cao, X., S.L. Pfaff, and F.H. Gage, *A functional study of miR-124 in the developing neural tube.* Genes Dev, 2007. **21**(5): p. 531-6.

59. Mishima, T., et al., *RT-PCR-based analysis of microRNA (miR-1 and -124) expression in mouse CNS.* Brain Res, 2007. **1131**(1): p. 37-43.

60. Song, Y., et al., *M2 microglia-derived exosomes protect the mouse brain from ischemia-reperfusion injury via exosomal miR-124.* Theranostics, 2019. **9**(10): p. 2910-2923.

61. Matsuoka, H., et al., *Levels of tight junction protein CLDND1 are regulated by microRNA-124 in the cerebellum of stroke-prone spontaneously hypertensive rats.* Biochem Biophys Res Commun, 2018. **498**(4): p. 817-823.

62. Saraiva, C., et al., *MicroRNA-124-loaded nanoparticles increase survival and neuronal differentiation of neural stem cells in vitro but do not contribute to stroke outcome in vivo.* PLoS One, 2018. **13**(3): p. e0193609.

63. Yang, J., et al., *Exosome Mediated Delivery of miR-124 Promotes Neurogenesis after Ischemia.* Mol Ther Nucleic Acids, 2017. **7**: p. 278-287.

64. Hamzei Taj, S., et al., *MiRNA-124 induces neuroprotection and functional improvement after focal cerebral ischemia.* Biomaterials, 2016. **91**: p. 151-165.

65. Pan, W.L., et al., *Ablation of the microRNA-17-92 cluster in neural stem cells diminishes adult hippocampal neurogenesis and cognitive function.* FASEB J, 2019. **33**(4): p. 5257-5267.

66. Jin, J., et al., *Distinct function of miR-17-92 cluster in the dorsal and ventral adult hippocampal neurogenesis.* Biochem Biophys Res Commun, 2018. **503**(3): p. 1594-1598.

67. Yang, P., et al., *The role of the miR-17-92 cluster in neurogenesis and angiogenesis in the central nervous system of adults.* J Neurosci Res, 2017. **95**(8): p. 1574-1581.

68. Abels, E.R. and X.O. Breakefield, *Introduction to Extracellular Vesicles: Biogenesis, RNA Cargo Selection, Content, Release, and Uptake.* Cell Mol Neurobiol, 2016. **36**(3): p. 301-12.

69. Frohlich, D., et al., *Multifaceted effects of oligodendroglial exosomes on neurons: impact on neuronal firing rate, signal transduction and gene regulation.* Philos Trans R Soc Lond B Biol Sci, 2014. **369**(1652).

70. Fruhbeis, C., et al., *Neurotransmitter-triggered transfer of exosomes mediates oligodendrocyte-neuron communication.* PLoS Biol, 2013. **11**(7): p. e1001604.

71. Kramer-Albers, E.M., et al., *Oligodendrocytes secrete exosomes containing major myelin and stress-protective proteins: Trophic support for axons?* Proteomics Clin Appl, 2007. **1**(11): p. 1446-61.

72. Han, Y., et al., *Multipotent mesenchymal stromal cell-derived exosomes improve functional recovery after experimental intracerebral hemorrhage in the rat.* J Neurosurg, 2018: p. 1-11.

73. Mahmood, A., D. Lu, and M. Chopp, *Marrow stromal cell transplantation after traumatic brain injury promotes cellular proliferation within the brain.* Neurosurgery, 2004. **55**(5): p. 1185-93.

74. Erdo, F., et al., *Host-dependent tumorigenesis of embryonic stem cell transplantation in experimental stroke.* J Cereb Blood Flow Metab, 2003. **23**(7): p. 780-5.

75. Matsumoto, J., et al., *The Transport Mechanism of Extracellular Vesicles at the Blood-Brain Barrier.* Curr Pharm Des, 2017. **23**(40): p. 6206-6214.

76. Alvarez-Erviti, L., et al., *Delivery of siRNA to the mouse brain by systemic injection of targeted exosomes.* Nat Biotechnol, 2011. **29**(4): p. 341-5.

77. Ortega, S.B., et al., *B cells migrate into remote brain areas and support neurogenesis and functional recovery after focal stroke in mice.* Proc Natl Acad Sci U S A, 2020.

78. Schaar, K.L., M.M. Brenneman, and S.I. Savitz, *Functional assessments in the rodent stroke model.* Exp Transl Stroke Med, 2010. **2**(1): p. 13.

79. Zhang, L., et al., *A test for detecting long-term sensorimotor dysfunction in the mouse after focal cerebral ischemia.* J Neurosci Methods, 2002. **117**(2): p. 207-14.

80. Tabak, S., S. Schreiber-Avissar, and E. Beit-Yannai, *Extracellular vesicles have variable dose-dependent effects on cultured draining cells in the eye.* J Cell Mol Med, 2018. **22**(3): p. 1992-2000.

Figure legends

Figure 1. Western blotting, nanosight tracking analysis (NTA), and transmission electron microscopy (TEM) of enriched NPC-EVs. Neural progenitor cells (NPCs) were cultured under standard cell culture conditions, and conditioned medium was obtained at passage 3 at 24 h after cell seeding. Conditioned medium was used for the enrichment of extracellular vesicles (EVs) using either the polyethylene glycol (PEG) method or differential centrifugation (i.e., ultracentrifugation). **(A)** Western blot analysis of EVs (n=4 per isolation conditions) against exosomal markers, with calnexin being used as a negative marker and ß-actin serving as a loading control. **(B)** Densitometric analysis from Western blot analysis from (A). **(C)** NTA from enriched EVs depicting size distribution patterns. **(D)** Representative TEM analysis from EVs enriched by either differential centrifugation (UC) or the PEG method. The negative control consisted of PBS only, depicting an artifact with no vesicular structure. Black arrows: exosomes; red arrows: microvesicles. Scale bar: 200 nm. Abbreviations: EV, extracellular vesicles; PBS, phosphate-buffered saline; PEG, polyethylene glycol; NPC, neural progenitor cell; NTA, nanosight tracking analysis; TEM, transmission electron microscopy; UC, ultracentrifugation.

Figure 2. **Mass spectrometric analysis and qRT-PCR analysis of selected proteins and miRNAs in NPC-derived EVs.** NPC-EVs were enriched using the PEG approach or ultracentrifugation as described before. Summed spectral counts of selected proteins from triplicate measurements are displayed. **(A)** Detection of cytosolic proteins including exosomal markers such as HSPA8, TSG101, and Alix. **(B)** Detection of transmembrane or GPI-anchored proteins in the two EV fractions. **(C)** Detection of intracellular proteins. Calnexin was found in both NPC-EV samples, although it is usually regarded as a negative control. Recent data, however, suggests that calnexin can also be found in several exosomal subtypes (see the appropriate results section). **(D)** Detection of exosomal negative markers APOA1, UMOD, and ALB. These markers were detected neither PEG-enriched nor in ultracentrifugation-enriched

EVs. **(E)** Screening for selected miRNAs using qRT-PCR in NPC-EVs enriched with the PEG method or with ultracentrifugation only. The results are presented as $2^{-\Delta Ct}$. Abbreviations: EV, extracellular vesicles; NPC, neural progenitor cell; PBS, phosphate-buffered saline; PEG, polyethylene glycol method; UC, ultracentrifugation only method.

Figure 3. NPC-EVs enhance the resistance of cerebral organoids exposed to oxygen-glucose-deprivation (OGD). Cerebral organoids were obtained and cultured under standard cell culture conditions, as explained in the materials and methods section. **(A)** Representative photos from EV-treated cerebral organoids, as described in (C). **(B)** The temporal resolution of the development of cell death under OGD conditions was determined for cerebral organoids (n=3 per condition) exposed to 8 h or 10 h of OGD, followed by reoxygenation under standard cell culture conditions for an additional 24 h. Cell death rates were assessed using TUNEL staining on cryostat sections, with DAPI staining for nuclear detection. **(C)** Since no statistical significance of cell death rates of organoids exposed to either 8 h or 10 h of OGD was observed, further experiments were done using the 8 h time window only. Organoids were treated with NPC-derived EVs at the beginning of the OGD and additionally at the beginning of the reoxygenation. Three different NPC-EVs concentrations were chosen (n=3 per condition), i.e., NPC-EVs low (EVs equivalent to 2×10^5 NPCs), NPC-EVs medium (EVs equivalent to 2×10^6 NPCs), and NPC-EVs high (EVs equivalent to 2×10^7 NPCs). Control organoids were exposed to OGD only without EV treatment, whereas “normal” refers to cerebral organoids kept under standard cell culture conditions. Scale bars: 20 µm. *significantly different from controls with $p<0.05$, i.e., NPC-EVs low: $p=0.0015$, NPC-EVs medium: $p=0.0015$, and NPC-EVs high: $p=0.0014$. $^{\#}$significantly different from standard cell culture conditions (normal) with $p<0.05$, i.e., OGD 8 h or OGD 10 h: $p<0.0001$, NPC-EVs low: $p<0.0001$, NPC-EVs medium: $p<0.0001$, NPC-EVs high: $p<0.0001$. Abbreviations: EV, extracellular vesicles; OGD, oxygen-glucose-deprivation; PBS, phosphate-buffered saline; NPC, neural progenitor cell; TUNEL, terminal deoxynucleotidyl transferase dUTP nick end labeling.

Figure 4. Delivery of NPC-EVs reduces post-ischemic motor coordination impairment. Motor coordination was evaluated using the balance beam test **(A)**, the tight rope test **(B)**, and the corner turn test **(C)** at 1, 7, 14, 28, 56, and 84 days after cerebral ischemia. All animals were accordingly trained before the induction of stroke in order to ensure proper test performance, i.e., test results before induction of stroke are given as pre-stroke data. Mice were exposed to 1 h of middle cerebral artery occlusion with subsequent reperfusion. Mice received systemic delivery of PBS (control, n=11), NPC-EVs low (EVs equivalent to $2x10^5$ NPCs, n=12), NPC-EVs medium (EVs equivalent to $2x10^6$ NPCs, n=11), or NPC-EVs high (EVs equivalent to $2x10^7$ NPCs, n=12), MSC-EVs low (EVs equivalent to $2x10^5$ MSCs, n=12), MSC-EVs medium (EVs equivalent to $2x10^6$ MSCs, n=12), or MSC-EVs high (EVs equivalent to $2x10^7$ MSCs, n=13) 24 h, 72 h and 120 h post-stroke. The first injections at 24 h were done via cannulation of the right femoral vein, whereas the following injections used a retroorbital injection delivery. All EV dosage groups showed significant improvement in the tight rope test compared to the control group (p<0.0001). In the corner turn test, the EV medium concentration group showed improvement at all experimental time points (p<0.0001 in NPC-EV medium group, p<0.0001 in MSC-EV medium group), and the EV high dosage group showed improvement at day 56 (p=0.0002 in NPC-EV high group, p=0.0004 in MSC-EV high group) and at day 84 (p<0.0001 in NPC-EV high group, p<0.0001 in MSC-EV high group) compared to the control group. In the balance beam test, however, only the EV medium group showed improvement at day 7 (p=0.0006 in NPC-EV medium group, p=0.0040 in MSC-EV medium group), day 14 (p=0.023 in NPC-EV medium group, p=0.0021 in MSC-EV medium group), and day 28 (p=0.0129 in NPC-EV medium group) compared to the control group. **(D)** Laser-doppler results of different experimental groups during MCAO operation. *significantly different from controls with p<0.05. Abbreviations: PBS, phosphate-buffered saline; MSC, mesenchymal stem cells; NPC, neural progenitor cell; EV, extracellular vesicles.

Figure 5. NPC-EVs induce long-term neuroprotection and increase cell proliferation after stroke. The neuronal density **(A)** and cell proliferation **(B)** were measured on day 84, as indicated by NeuN staining **(A)** within the ischemic lesion site and cell proliferation, as indicated by BrdU staining **(B)** in order to investigate. The extent of neurogenesis was analyzed on day 84, as indicated by the neuronal marker NeuN **(C)** and the immature neuronal marker Dcx **(D)**. Co-immunofluorescence staining was done with the cell proliferation marker BrdU. Mice were exposed to 60 min of focal cerebral ischemia and treated with EVs at different dosages. The first injections at 24 h were done via cannulation of the right femoral vein, whereas the following injections used a retroorbital injection delivery. Mice were exposed to middle cerebral artery occlusion followed by systemic delivery of PBS (control, n=11), NPC-EVs low (EVs equivalent to $2x10^5$ NPCs, n=12), NPC-EVs medium (EVs equivalent to $2x10^6$ NPCs, n=11), or NPC-EVs high (EVs equivalent to $2x10^7$ NPCs, n=12), MSC-EVs low (EVs equivalent to $2x10^5$ MSCs, n=12), MSC-EVs medium (EVs equivalent to $2x10^6$ MSCs, n=12), or MSC-EVs high (EVs equivalent to $2x10^7$ MSCs, n=13) 24 h, 72 h and 120 h post-stroke. Photos depict representative stainings from the respective condition. Both NPC-EV medium and MSC-EV medium groups increased neuronal density, as shown in (A; p=0.0007 in NPC-EV medium group, p=0.0022 in MSC-EV medium group) and stimulated endogenous cell proliferation (B; p<0.0001 in NPC-EV medium group, p<0.0001 in MSC-EV medium group). Newborn neurons were also increased in NPC-EV medium and MSC-EV medium groups (C; p=0.0015 in NPC-EV medium group, p=0.0037 in MSC-EV medium group). The NPC-EV medium and MSC-EV medium group also showed higher cell proliferation levels compared to NPC-EV or MSC-EV high or low dosage groups (B; p<0.0001 in NPC-EV medium group, p<0.0001 in MSC-EV medium group compared to the low or high dosage NPC-EV or MSC-EV groups). However, there was no significant difference between the NPC-EV medium group and the MSC-EV medium group. Scale bars: 20 µm. *significantly different from controls with p<0.05. $^{\#}$significantly different from low or high dosage NPC-EV or MSC-EV groups with p<0.05. Abbreviations: BrdU, 5-Bromo-2-deoxyuridine; DAPI, 4’,6-diamidino-2-phenylindole;

Dcx, doublecortin; EV, extracellular vesicles; MSC, mesenchymal stem cells; NPC, neural progenitor cell; PBS, phosphate-buffered saline.

Figure 6. NPC-EV delivery affects post-ischemic axonal plasticity. Mice were exposed to 60 min of focal cerebral ischemia, as mentioned previously. Animals received systemic delivery of PBS (control, n=11), NPC-EVs low (EVs equivalent to $2x10^5$ NPCs, n=12), NPC-EVs medium (EVs equivalent to $2x10^6$ NPCs, n=11), or NPC-EVs high (EVs equivalent to $2x10^7$ NPCs, n=12), MSC-EVs low (EVs equivalent to $2x10^5$ MSCs, n=12), MSC-EVs medium (EVs equivalent to $2x10^6$ MSCs, n=12), or MSC-EVs high (EVs equivalent to $2x10^7$ MSCs, n=13) 24 h, 72 h and 120 h post-stroke. The first injections at 24 h were done via cannulation of the right femoral vein, whereas the following injections used a retroorbital injection delivery. On day 70 after stroke induction, biotinylated dextran amine (BDA) was injected using a Hamilton syringe at 0.5 mm rostral from bregma, 2.5 mm lateral from bregma, and 1.5 mm ventral from the cortical surface. Axonal density was measured 84 days post-stroke, i.e., 14 days after injection of BDA. There was a significant difference in the EV medium and EV high dosage (NPC-EVs only) groups compared to the control group (p=0.0049 in NPC-EV medium group, p=0.0331 in NPC-EV high group, p=0.0075 in MSC-EV medium group). Scale bars: 200 µm. *significantly different from control with p<0.05. Abbreviations: EV, extracellular vesicles; MSC, mesenchymal stem cells; NPC, neural progenitor cell; PBS, phosphate-buffered saline.

Figure 7. NPC-EVs reverse post-ischemic peripheral immunosuppression at 7 days after ischemia. Mice were subjected to cerebral ischemia for 60 min followed by reperfusion for 7 days. The animals (n=5 per condition) received systemic delivery of PBS (control), NPC-EVs low (EVs equivalent to $2x10^5$ NPCs), NPC-EVs medium (EVs equivalent to $2x10^6$ NPCs), or NPC-EVs high (EVs equivalent to $2x10^7$ NPCs) 24 h, 72 h and 120 h post-stroke. Flow cytometry was analyzed with FlowJo software. **(A-D)** show the quantitative analysis from CNS samples for **(A)** $CD45^+$ int^-, **(B)** $CD11b^+$, **(C)** $CD3^+$, and **(D)** $CD19^+$ cells. In **(E-H)**, the

quantitative analyses from blood samples for **(E)** CD45$^+$ int$^-$, **(F)** CD11b$^+$, **(G)** CD3$^+$, and **(H)** CD19$^+$ cells is given. NPC-EV medium increased both T cell (*p*=0.0014) and B cell populations (*p*=0.0015) in the peripheral blood but did not affect the CNS. All data are given as means ± S.D., *significantly different from control, *p*<0.05. Abbreviations: CNS, central nervous system; EV, extracellular vesicles; NPC, neural progenitor cell.

Figure 8. Biodistribution patterns of NPC-EVs in vivo. Representative immunofluorescence images displaying the biodistribution of EVs in various organs under different delivery routes and conditions (femoral vein injection, femoral vein injection after MCAO, retroorbital injection, and retroorbital injection after MCAO). The organs selected for qualitative and quantitative analysis included the brain, liver, and lung (red color shows the DiI marker for EV detection). Most EVs were found in the liver (*p*=0.0023 compared with the brain) and in the lung (*p*=0.0035 compared with the brain). However, the NPC-EV biodistribution patterns were of no difference between the liver and the lung. Different delivery routes and conditions also did not affect the biodistribution of NPC-EVs in peripheral organs. The representative photographs refer to mice exposed to MCAO followed by femoral vein injection of EVs. Scale bars: 200 µm. Data are shown as mean ± S.D; *significantly different from the brain, p<0.05. n=3 animals per group. Abbreviations: EVs, extracellular vesicles; MCAO, middle cerebral artery occlusion; NPC, neural progenitor cell.

Figure.1

Click here to access/download;Figure;Figure_1_revised.tiff

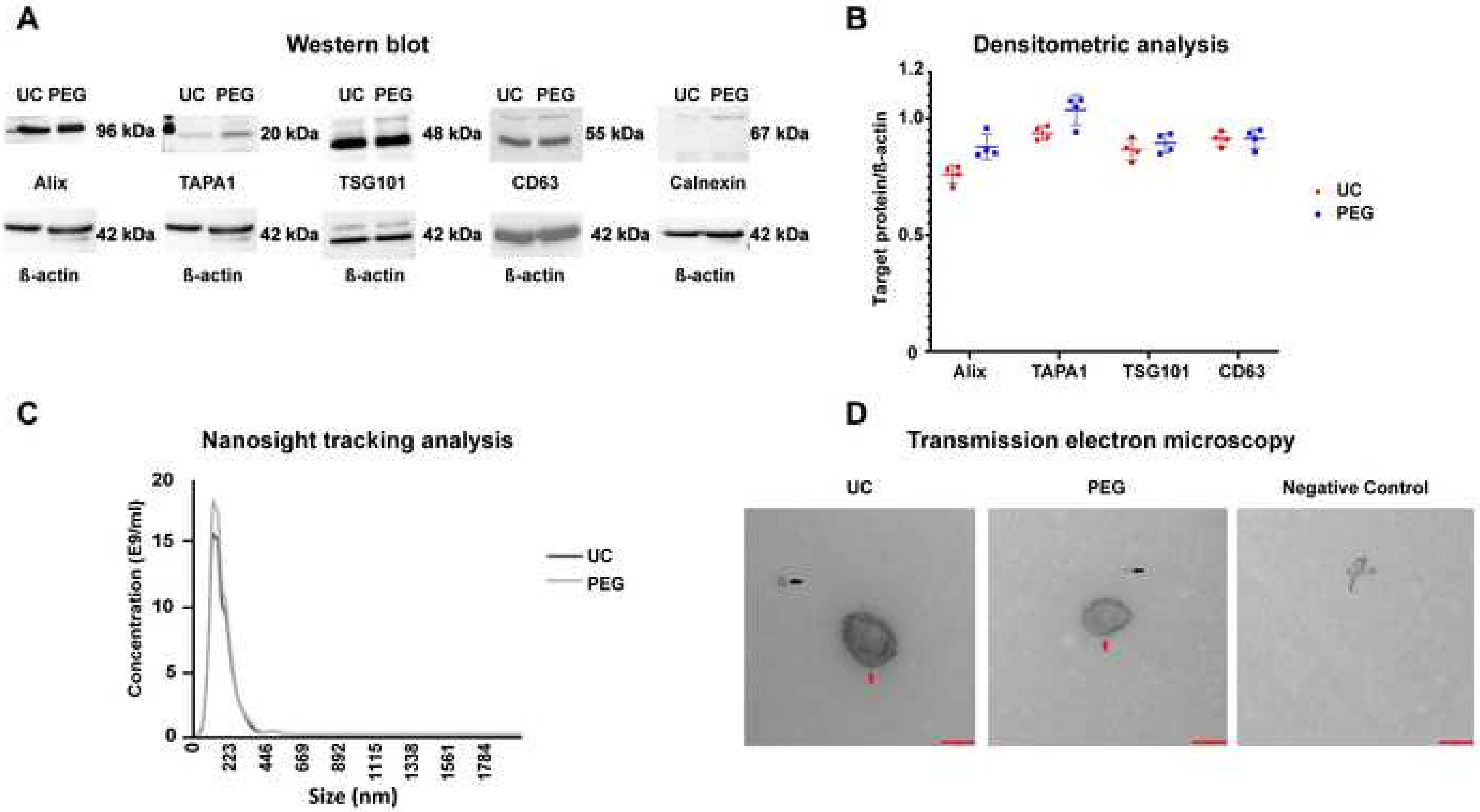

Figure.2

Click here to access/download;Figure;Figure_2_revised.tiff

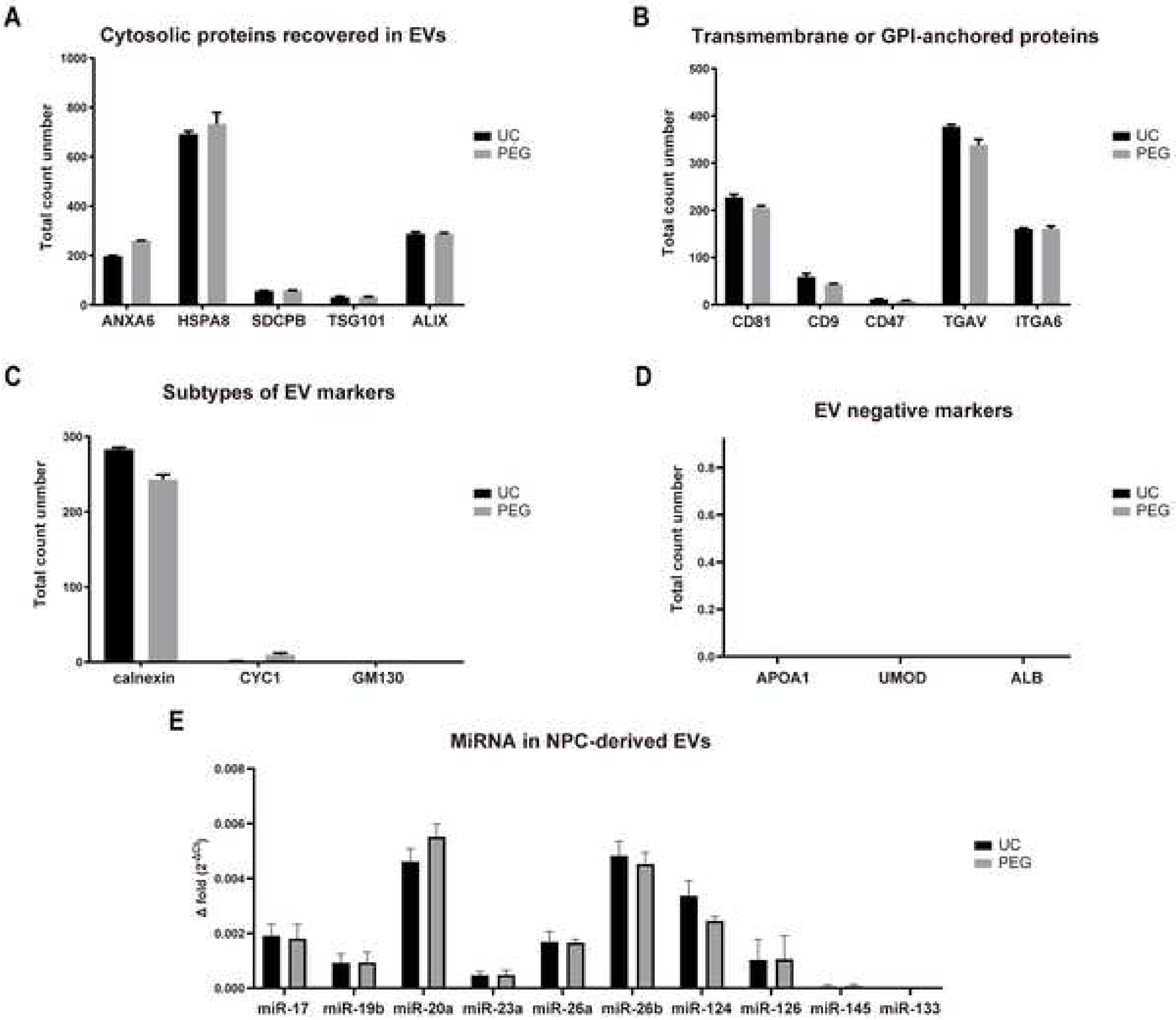

Figure.3

Click here to access/download;Figure;Figure_3_revised.tiff

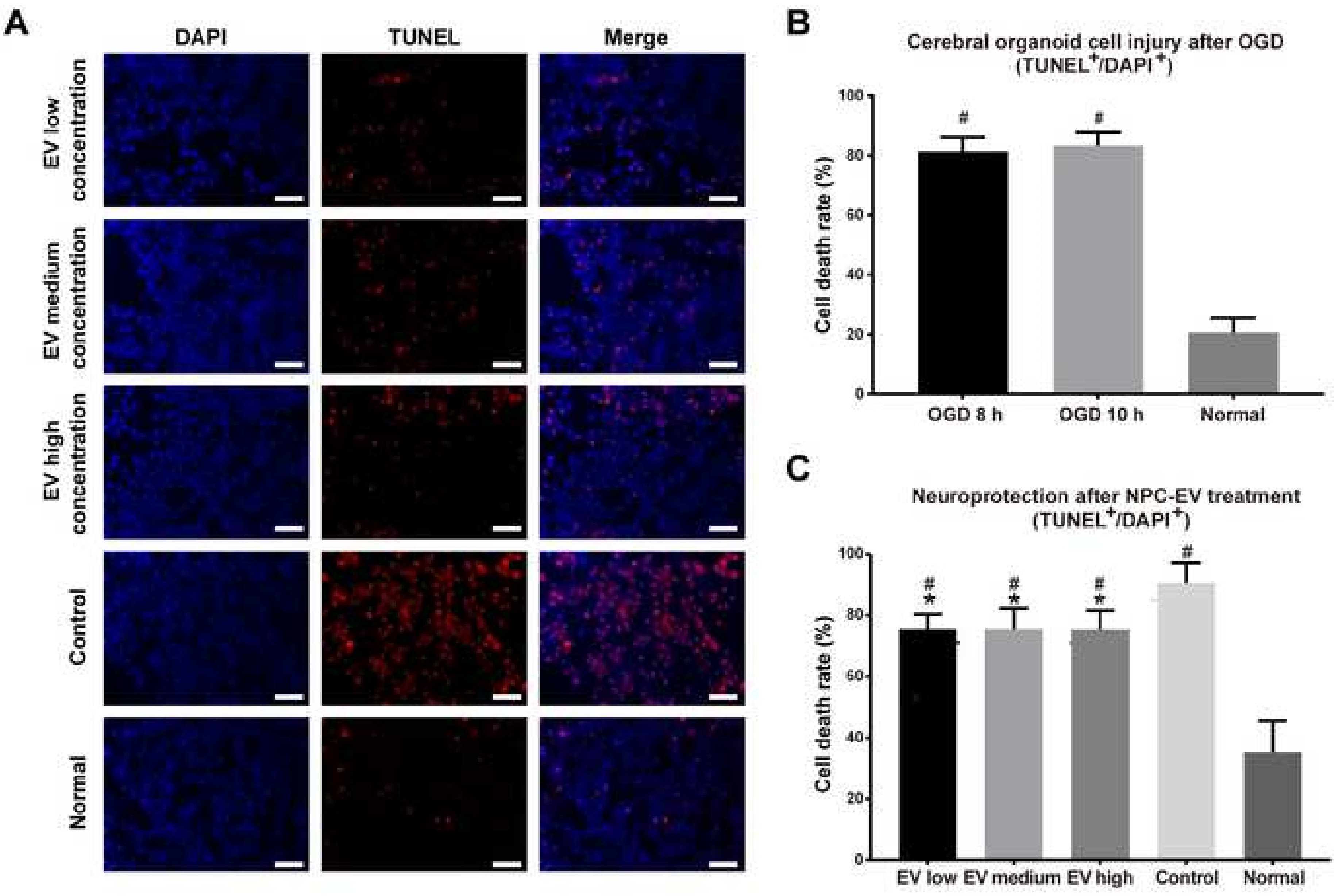

Figure.4

Click here to access/download;Figure;Figure_4_revised.tiff

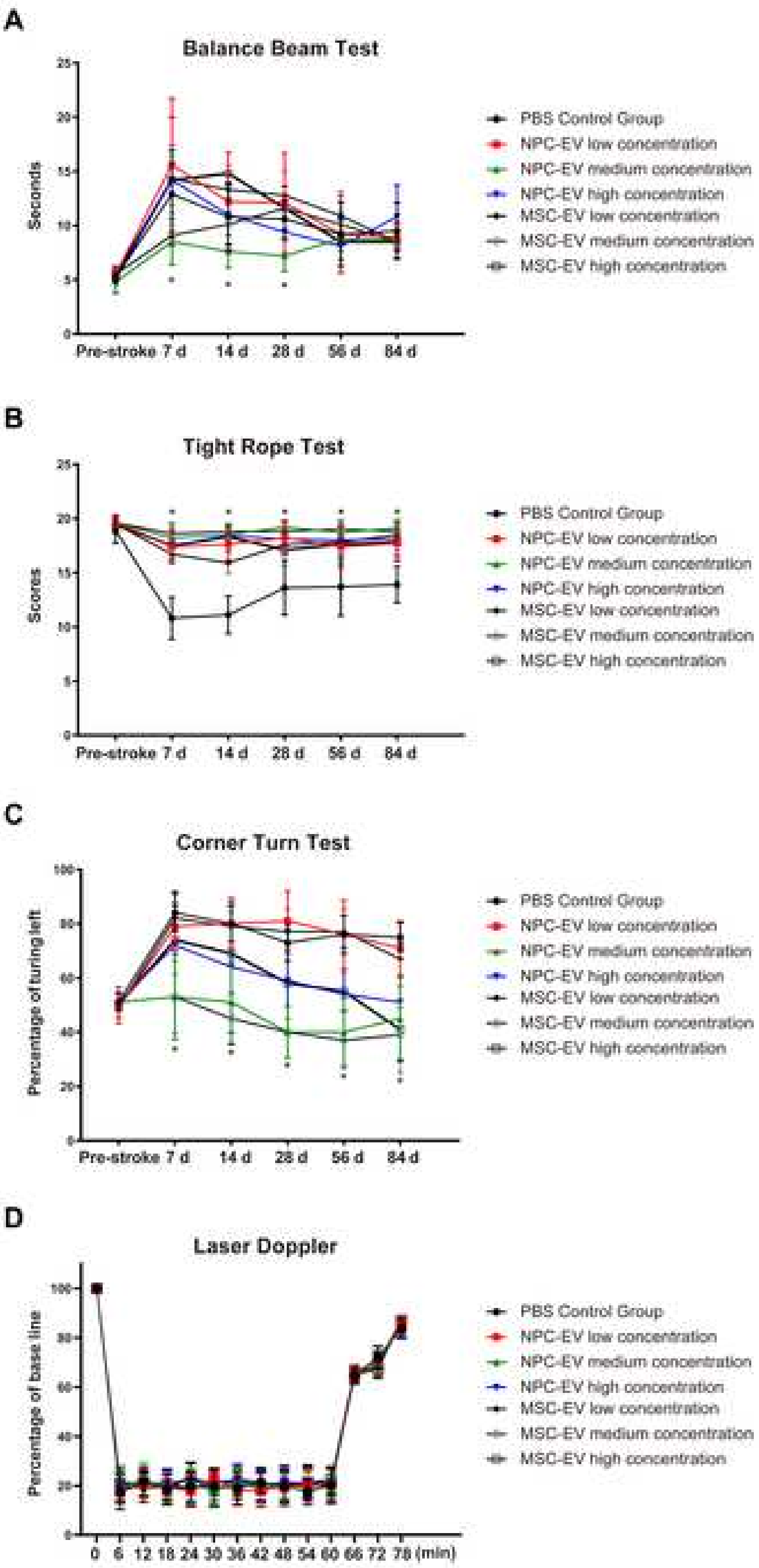

Figure.5

Click here to access/download;Figure;Figure_5_revised.tiff

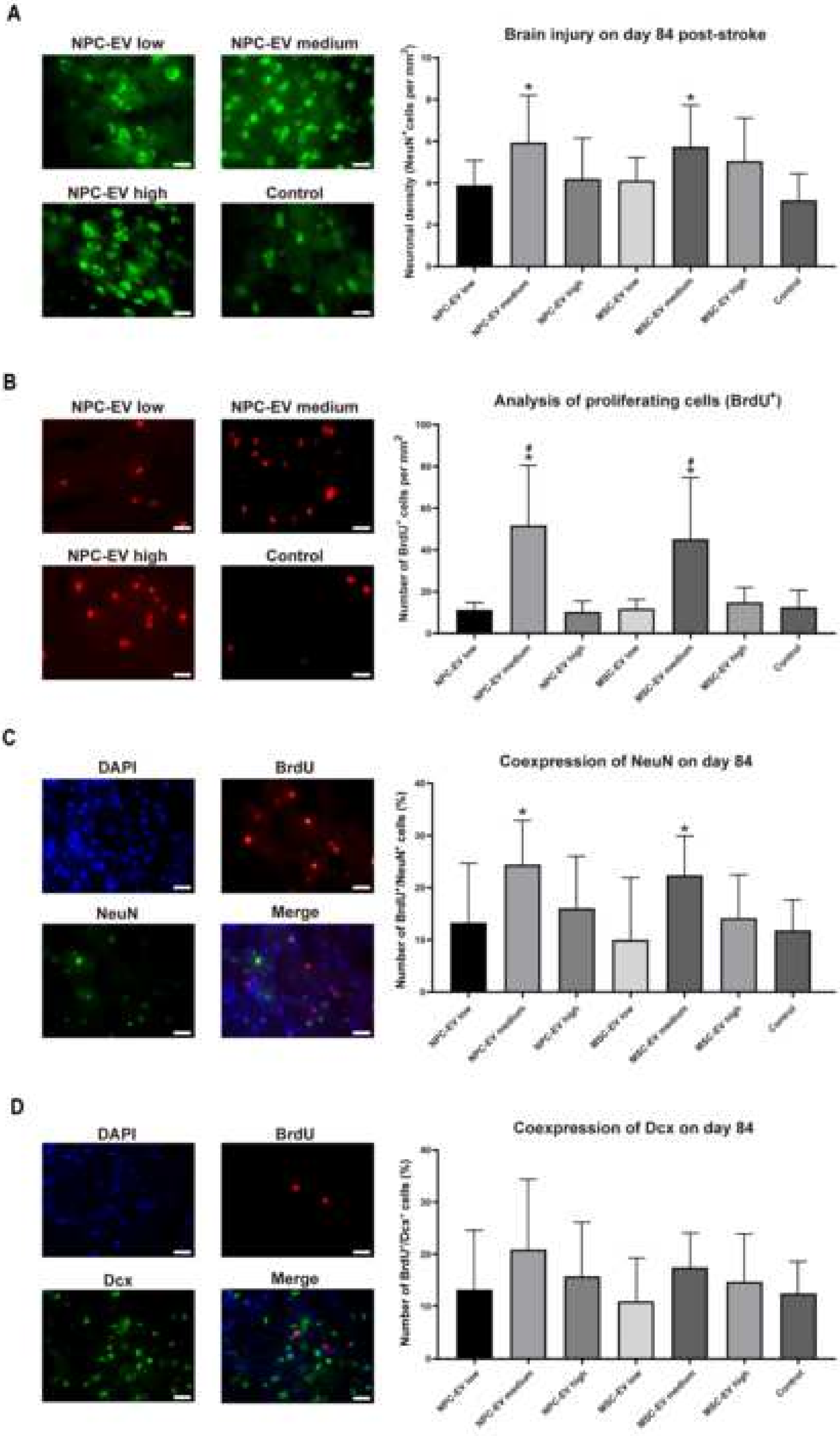

Click here to access/download;Figure;Figure_6_revised.tiff

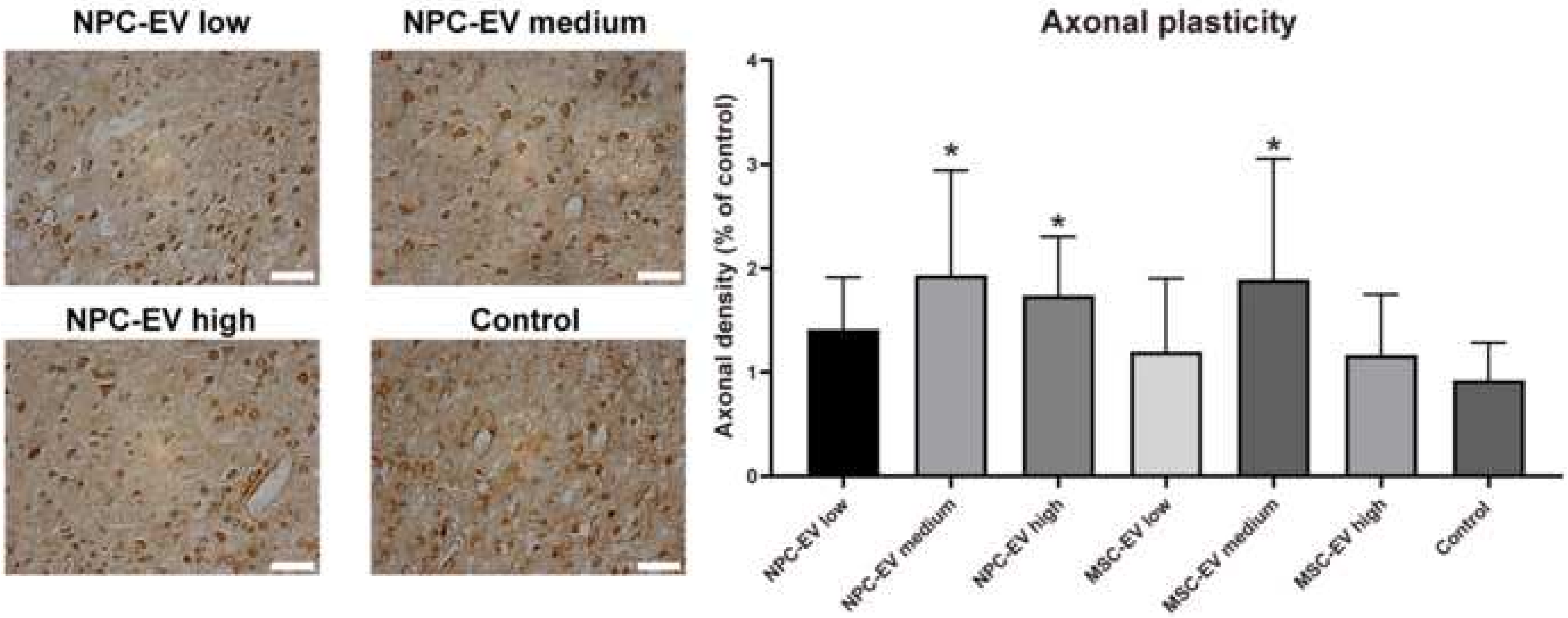

Figure.7

Click here to access/download;Figure;Figure_7_revised.tiff

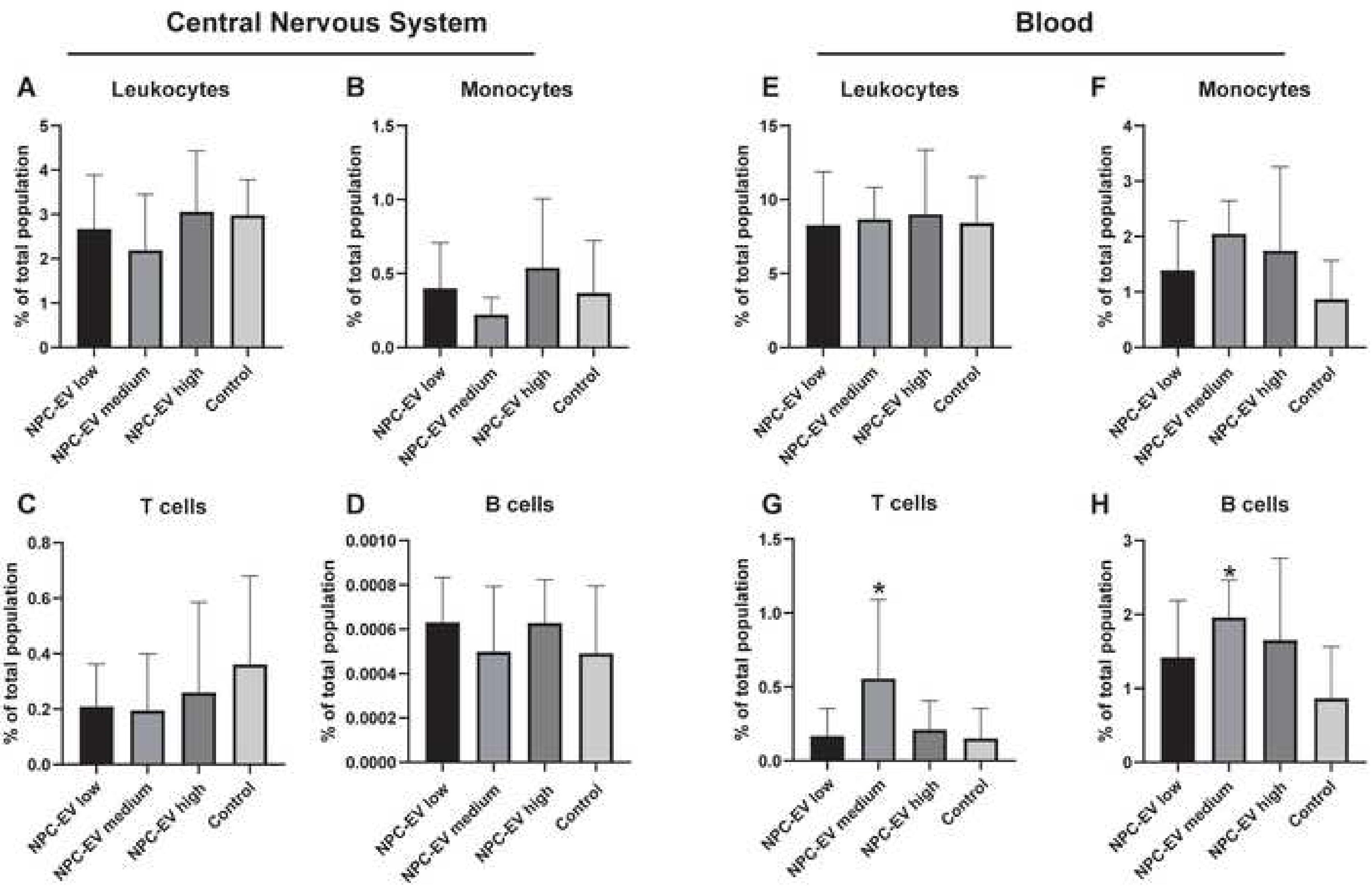

Figure.8

Click here to access/download;Figure;Figure_8_revised.tiff

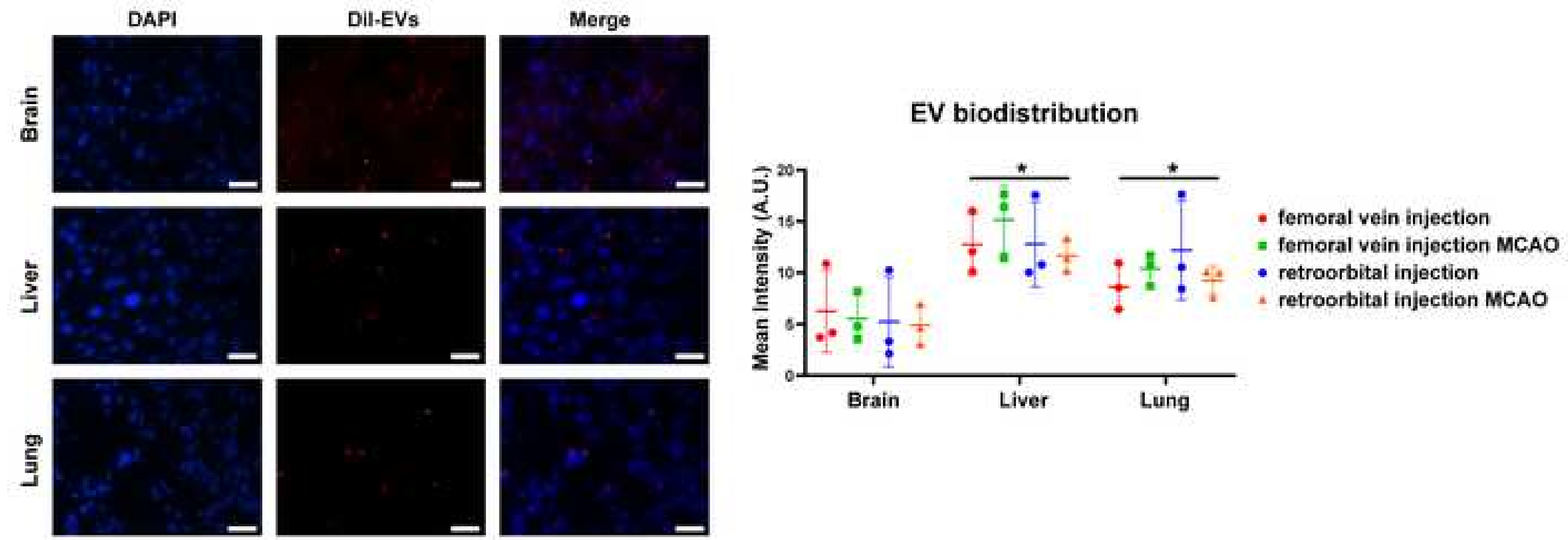

Click here to access/download
Supplementary Material
mass spectrometric analyses.xlsx

Click here to access/download
Supplementary Material
Supplementary.pdf
